# Dimethyl fumarate-related immune and transcriptional signature is associated with clinical response in multiple sclerosis-treated patients

**DOI:** 10.3389/fimmu.2023.1209923

**Published:** 2023-07-07

**Authors:** Alicia Sánchez-Sanz, Santiago García-Martín, Julia Sabín-Muñoz, Irene Moreno-Torres, Víctor Elvira, Fátima Al-Shahrour, Aranzazu García-Grande, Elvira Ramil, Ofir Rodríguez-De la Fuente, Beatriz Brea-Álvarez, Ruth García-Hernández, Antonio García-Merino, Antonio José Sánchez-López

**Affiliations:** ^1^ Neuroimmunology Unit, Instituto de Investigación Sanitaria Puerta de Hierro-Segovia de Arana, Madrid, Spain; ^2^ PhD Program in Molecular Biosciences, Doctoral School, Universidad Autónoma de Madrid, Madrid, Spain; ^3^ Bioinformatics Unit, Spanish National Cancer Research Centre (CNIO), Madrid, Spain; ^4^ Department of Neurology, Hospital Universitario Puerta de Hierro Majadahonda, Madrid, Spain; ^5^ Demyelinating Diseases Unit, Hospital Universitario Fundación Jiménez Díaz, Madrid, Spain; ^6^ School of Mathematics, University of Edinburgh, Edinburgh, United Kingdom; ^7^ Flow Cytometry Core Facility, Instituto de Investigación Sanitaria Puerta de Hierro-Segovia de Arana, Madrid, Spain; ^8^ Sequencing Core Facility, Instituto de Investigación Sanitaria Puerta de Hierro-Segovia de Arana, Madrid, Spain; ^9^ Radiodiagnostic Division, Hospital Universitario Puerta de Hierro Majadahonda, Madrid, Spain; ^10^ Department of Medicine, Universidad Autónoma de Madrid, Madrid, Spain; ^11^ Red Española de Esclerosis Múltiple (REEM), Barcelona, Spain; ^12^ Biobank, Instituto de Investigación Sanitaria Puerta de Hierro-Segovia de Arana, Madrid, Spain

**Keywords:** multiple sclerosis, dimethyl fumarate, biomarker, immunophenotype, transcriptome, disease-modifying therapies, treatment response, NEDA

## Abstract

**Background and objective:**

Dimethyl fumarate (DMF) is an immunomodulatory drug approved for the therapy of multiple sclerosis (MS). The identification of response biomarkers to DMF is a necessity in the clinical practice. With this aim, we studied the immunophenotypic and transcriptomic changes produced by DMF in peripheral blood mononuclear cells (PBMCs) and its association with clinical response.

**Material and methods:**

PBMCs were obtained from 22 RRMS patients at baseline and 12 months of DMF treatment. Lymphocyte and monocyte subsets, and gene expression were assessed by flow cytometry and next-generation RNA sequencing, respectively. Clinical response was evaluated using the composite measure “no evidence of disease activity” NEDA-3 or “evidence of disease activity” EDA-3 at 2 years, classifying patients into responders (n=15) or non-responders (n=7), respectively.

**Results:**

In the whole cohort, DMF produced a decrease in effector (TEM) and central (TCM) memory T cells in both the CD4+ and CD8+ compartments, followed by an increase in CD4+ naïve T cells. Responder patients presented a greater decrease in TEM lymphocytes. In addition, responder patients showed an increase in NK cells and were resistant to the decrease in the intermediate monocytes shown by non-responders. Responder patients also presented differences in 3 subpopulations (NK bright, NK dim and CD8 TCM) at baseline and 4 subpopulations (intermediate monocytes, regulatory T cells, CD4 TCM and CD4 TEMRA) at 12 months. DMF induced a mild transcriptional effect, with only 328 differentially expressed genes (DEGs) after 12 months of treatment. The overall effect was a downregulation of pro-inflammatory genes, chemokines, and activators of the NF-kB pathway. At baseline, no DEGs were found between responders and non-responders. During DMF treatment a differential transcriptomic response was observed, with responders presenting a higher number of DEGs (902 genes) compared to non-responders (189 genes).

**Conclusions:**

Responder patients to DMF exhibit differences in monocyte and lymphocyte subpopulations and a distinguishable transcriptomic response compared to non-responders that should be further studied for the validation of biomarkers of treatment response to DMF.

## 1 Introduction

Multiple sclerosis (MS) is an autoimmune disease of the central nervous system (CNS) characterized by an attack of the myelin sheath that surrounds and protects CNS axons, followed by axonal destruction and neuronal death. The pathophysiology of MS is complex and includes the infiltration into the CNS, through a leaked blood-brain barrier, of circulating inflammatory cells, including CD4+ and CD8+ T lymphocytes, B lymphocytes and macrophages. These cells, together with CNS-resident glial cells, lead to inflammation, demyelination and neurodegeneration (1).

Although there is no cure for MS, therapeutic management aims to slow disease progression and to prevent relapses and clinical disability (2). In this regard, a growing number of disease modifying therapies (DMTs) have been developed to date, all of them primarily targeting the peripheral immune system (3). Among them, dimethyl fumarate (DMF) (Tecfidera^®^, Biogen; also known as BG-12) has been approved for the treatment of relapsing-remitting multiple sclerosis (RRMS) (4). DMF is one of the most prescribed first-line DMTs due to its oral administration, which promotes therapeutic adherence, and to a favorable benefit-risk profile (5, 6). However, a variable number of patients exhibit recurrence or ongoing inflammatory activity, indicating that there may be a suboptimal response to DMF which ultimately results in a treatment switch (7).

While the molecular mechanism of action of DMF remains to be fully elucidated, several studies have shown that DMF affects mainly memory T and B cells and induces an anti-inflammatory shift in peripheral blood mononuclear cells (PBMCs) (8). DMF reduces the proportions of both effector memory and central memory T cells, whereas naïve T cells are increased in MS-treated patients (9–12). A similar effect is found in B lymphocytes, with a reduction in circulating memory B cells (11, 13, 14). Th1 and Th17 subsets are also diminished in patients treated with DMF, in contrast to an expansion of the Th2 subset (10, 12). However, the number of studies correlating changes in PBMCs subpopulations after DMF treatment with clinical response is still scarce. In this respect, associations with optimal response to DMF have been found between different immune populations such as CD56bright NK cells (11), monocytes (15) and IL-17 producing CD8+ T lymphocytes (16).

However, the use of predictive biomarkers of clinical outcomes is still limited in the clinical practice due to the heterogeneous results between studies (11, 15, 16). The combination of several laboratory markers could be a more reliable strategy to predict the clinical response in MS. In this regard, we have previously demonstrated that the combination of lymphocyte markers and gene expression profiling is useful for predicting clinical response in MS patients treated with fingolimod (17).

To deepen our knowledge of the immunomodulatory effects of DMF, we carried out a study of the cellular and transcriptional changes that occur in PBMCs of MS patients treated with DMF. In addition, we did a 2-year follow up of patients and classified them as responders or non-responders to DMF according to clinical response, measured by the “No evidence of disease activity” (NEDA) status. With the aim of identifying treatment response biomarkers, we correlated the cellular and transcriptional changes observed in PBMCs with the clinical response.

## 2 Methods

### 2.1 Patients and healthy donors

Enrolment was limited to patients with a diagnosis of RRMS according to McDonald criteria (18) and with an indication for treatment with DMF (4). Exclusion criteria included having received steroid treatment in the last month or any kind of immunosuppressants in the last year. A total of 22 patients were recruited from the Multiple Sclerosis Unit at Hospital Universitario Puerta de Hierro Majadahonda (HUPHM). Patients were treated with 240 mg DMF twice a day, according to the current recommendations of use for DMF (4). Venous blood samples were collected in tubes containing lithium heparin (Greiner Bio-One) immediately before starting treatment with DMF and 12 months after initiating therapy. Samples from 10 age and sex-matched healthy donors (HD) at a single time point were also included in the study. **Table 1** summarizes the demographic characteristics and the clinical data at baseline from the MS patients and HD in this study.

**Table 1:** Demographic characteristics of healthy donors and multiple sclerosis patients at baseline.

	Flow cytometry			
	Groups		MS Subgroups	
	HD (n=10)	MS (n=22)	NEDA-3 (n=15)	EDA-3 (n=7)
**Age (years)†**	43.60 ± 10.38	39.45 ± 9.60	40.73 ± 8.38	36.71 ± 12.08
**Sex (% of female)‡**	70%	77.27%	80%	71.43%
**Disease duration (years)†, §**	–	6.38 ± 6.66	7.49 ± 6.63	3.98 ± 6.56
**Time since DMT onset (years)†**	–	4.04 ± 5.57	4.44 ± 5.31	3.18 ± 6.44
**Number of previous DMTs†**	–	0.86 ± 1.08	0.67 ± 0.49	1.29 ± 1.80
Immediately previous treatment				
•Naïve:	–	36.36%	33.33%	42.86%
•Interferon:	–	54.55%	60%	42.86%
•Natalizumab:	–	4.55%	–	14.29%
•Glatiramer acetate:	–	4.55%	6.67%	–
Reason for treatment change				
•Naïve	–	36.36%	33.33%	42.86%
•Efficacy	–	18.18%	20%	14.29%
•Tolerability	–	45.45%	46.67%	42.86%
**Basal ARR†, ¶**	–	0.68 ± 0.68	0.56 ± 0.73*	0.93 ± 0.49*
**Basal EDSS†**	–	1.57 ± 1.33	1.30 ± 0.90	2.14 ± 1.93
**Number of GdE lesions†**	–	0.23 ± 0.53	0.13 ± 0.35	0.43 ± 0.79
**Number of new T2w lesions†, ¥**	–	1.29 ± 0.47	1.20 ± 0.42	1.50 ± 0.58
	RNA-seq			
	Groups		MS Subgroups	
	HD (n=10)	MS (n=10)	NEDA-3 (n=5)	EDA-3 (n=5)
**Age (years)†**	43.30 ± 10.60	39.90 ± 10.09	42.20 ± 8.87	37.60 ± 11.72
**Sex (% of female)‡**	70%	80%	80%	80%
**Disease duration (years)†, §**	–	5.39 ± 6.10	5.42 ± 5.27	5.37 ± 7.49
**Time since DMT onset (years)†**	–	4.05 ± 5.64	3.73 ± 3.93	4.37 ± 7.49
**Number of previous DMTs†**	–	1.10 ± 1.52	0.60 ± 0.55	1.60 ± 2.07
Immediately previous treatment				
•Naïve	–	40%	40%	40%
•Interferon	–	40%	40%	40%
•Natalizumab	–	10%	–	20%
•Glatiramer acetate	–	10%	20%	–
Reason for treatment change				
•Naïve	–	40%	40%	40%
•Efficacy	–	30%	40%	20%
•Tolerability	–	30%	20%	40%
**Basal ARR†, ¶**	–	0.83 ± 0.81	0.96 ± 1.19	0.70 ± 0.11
**Basal EDSS†**	–	1.95 ± 1.72	1.10 ± 1.14	2.80 ± 1.89
**Number of GdE lessions†**	–	0.10 ± 0.32	0.20 ± 0.45	0.00 ± 0.00
**Number of new T2w lesions†, ¥**	–	1.33 ± 0.52	1.00 ± 0.00	1.67 ± 0.58

Flow cytometry experiments were performed in samples from 22 multiple sclerosis (MS) patients and 10 healthy donors (HD). RNA-seq experiments were performed in samples from 10 MS patients and 10 HD. In both experiments, MS patients were classified according to their clinical response to dimethyl fumarate at 2 years, into no evidence of disease activity 3 (NEDA-3) or evidence of disease activity 3 (EDA-3).

†Values are the mean ± standard deviation (SD) of each group. The Mann-Whitney test was used to compare differences between MS and HD, and between NEDA-3 and EDA-3 MS patients.

‡Chi-Square test was used to compare two proportions.

§Time since the first symptoms of MS.

¶For Basal annualized relapse rate (ARR), only the previous year was considered.

¥Data not available for naïve MS patients, as most of them had a single magnetic resonance imaging (MRI).

*p<0.05

DMT, disease-modifying treatment; EDSS, expanded disability status scale; GdE, gadolinium-enhanced T1 lesions; T2w, T2-weighted lesions

### 2.2 Clinical response and MRI measures

Expanded disability status scale (EDSS) and clinical relapses were evaluated in all patients at baseline, 12 and 24 months after starting therapy with DMF. A 1.5T brain magnetic resonance imaging (MRI) was performed at the same time points to measure the number of gadolinium-enhanced T1 lesions (GdE) and the number of new or enlarged T2-weighted lesions (T2w). MRI activity was defined as the appearance of new GdE and/or T2w lesions. Confirmed disability progression (CDP) was defined as an increase of at least 0.5 EDSS points sustained for three months. NEDA-3 was calculated according to published parameters (no MRI activity, no relapses, and no CDP) (19). Patients were classified as DMF responders or non-responders according to their NEDA-3 status at 2 years.

### 2.3 PBMCs isolation and flow cytometry

PBMCs were isolated from peripheral blood, immediately as it was collected, by Ficoll density gradient centrifugation and cryopreserved in liquid nitrogen until use. Prior to staining, cells were pre-incubated with FcR Blocking Reagent (Miltenyi Biotec). PBMCs were stained with fluorochrome-conjugated antibodies (FCAbs) against human cell surface and intracellular markers, defining 54 monocyte and lymphocyte subsets (**Supplementary Table 1**). Gating strategy is shown in **Supplementary Figure 1**. For intracellular cytokine staining, PBMCs were stimulated for 4 hours with 100 ng/ml of phorbol 12-myristate 13-acetate (PMA) and 1 μg/ml ionomycin in the presence of 10 μg/ml brefeldin A (all from Sigma-Aldrich). Cells were fixed and permeabilized using INTRACELL-Kit (Immunostep). To exclude dead cells from the analysis, 4’,6-diamidine-2-phenylindole (DAPI) was used in the surface staining panels and the LIVE/DEAD™ Fixable Violet Dead Cell Stain Kit (Thermo Fisher Scientific) was used in the intracellular staining panels. All FCAbs were purchased from Miltenyi Biotec. Data were acquired using a digital flow cytometer (MACSQuant 10, Miltenyi Biotec) and analyzed using MACSQuantify (Miltenyi Biotec) and FlowJo (BD Biosciences) software.

### 2.4 Statistical analysis

Statistical analyses were performed using Prism 8 (GraphPad) and STATA 13 (StataCorp) software. The Mann-Whitney test was used for comparisons between HD and patients at baseline and between subgroups (responder vs. non-responder, women vs. men, or naïve vs. previously treated). The Wilcoxon signed-rank test was used for paired samples between baseline and 1 year. Statistical significance was established at p<0.05. Because the research was designed as a discovery study, p-values were not adjusted to maximize the finding of new biomarkers and the generation of new hypotheses.

### 2.5 RNA isolation and sequencing (RNA-seq)

Total RNA from PBMCs was extracted using the Maxwell 16 LEV simply RNA Cells kit (Promega Biotech Ibérica) on the Maxwell 16 instrument, according to the manufacturer’s instructions. For RNA-seq experiments, we selected 10 HD and 10 MS patients (5 responders and 5 non-responders) at baseline and after 1 year of DMF treatment, from the whole cohort of patients. The demographic characteristics of the sequenced HD and MS patients were similar to those of the whole sample and are summarized in **Table 1**. The quality and quantity of the RNA was determined in Bioanalyzer 2100 (Agilent Technologies) and Qubit 3.0 (Thermo Fisher Scientific), respectively. Poly(A)+ mRNA fraction was isolated from total RNA and cDNA libraries were obtained following Illumina’s recommendations. Briefly, poly(A)+ RNA was isolated on poly-T oligo-attached magnetic beads and chemically fragmented prior to reverse transcription and cDNA generation. The cDNA went through an end repair process, addition of a single “A” base to the 3’ end and ligation to the adapters. Finally, the products were purified and enriched with PCR to create the indexed final double stranded DNA library. The quality and quantity of the libraries were analyzed in tapeStation 4200, using the High Sensitivity assay (Agilent Technologies). The pool of libraries was sequenced by pair-end sequencing (150x2) in Novaseq 6000 (Illumina) at a sequencing depth of 40 million reads per sample.

### 2.6 Bioinformatics analysis

Quality control of the raw sequencing reads was performed with FastQC v.0.11.9. Adapter sequences were trimmed with cutadapt v2.10. Trimmed reads were quantified with Salmon v.1.3.0 (20) and summarized to the gene level with tximeta v.1.6.3 (21). Salmon was used in selective alignment mode with the entire human genome as a decoy sequence.

All the differential gene expression analyses were performed with DESeq2 v1.28 (22) and R v4.0.2. Only genes with p-value<0.05 after the false discovery rate (FDR) correction were considered statistically significant. For the comparisons between HD and MS patients at baseline and after 1 year of DMF treatment, we fitted a linear model of the treatment condition with each patient’s phenotypic sex as a covariate. Likewise, the transcriptomic differences between responders, non-responders and HD after DMF treatment were modelled as a multi-factor design with the case id as a covariate in the design formula.

## 3 Results

### 3.1 Clinical and radiological response to DMF treatment

At baseline (**Table 1**), the mean age of our cohort of patients was 39 years, with a disease duration of 6 years. Nearly 80% (n= 17) were female. About 36% were treatment naïve (n= 8), and previously treated patients had received interferon beta (n=12), natalizumab (n=1) or glatiramer acetate (n=1). The patient who was switched from natalizumab started DMF due to tolerability issues. Around 45% (n= 10) had switched to DMF for tolerability, and 18% (n=4) due to lack of efficacy.

**Table 2** summarizes the clinical and MRI measures obtained at 1 and 2 years of treatment with DMF. In our cohort, DMF reduced the mean annualized relapse rate (ARR) at 1 and 2 years compared to baseline and almost all patients (n=21) were relapse-free during follow-up. The mean EDSS remained stable at 2 years. At one year all patients were free of CDP, while at 2 years only 86% (n=19) were free of CDP. The number of T2w lesions was reduced at 1 and 2 years, and only 10% (n=2) of the patients presented new T2w lesions at 1 and 2 years. None of the patients presented GdE lesions at one year, although at 2 years 9% (n=2) of the patients developed new GdE lesions. The percentage of NEDA-3 patients was 86% (n=18) at 1 year and 68% (n=15) at 2 years. None of the patients discontinued DMF before the end of the 2-year follow-up.

**Table 2:** Clinical and radiological response to DMF treatment.

	Time periods			Statistical significance		
	Baseline	1 year	2 years	Baseline vs 1 year	1 year vs 2 years	Baseline vs 2 years
**ARR†, ‡**	0.68 ± 0.68	0.05 ± 0.21	0.05 ± 0.15	**p<0.0001**	p>0.9999	**p<0.0001**
**EDSS†**	1.57 ± 1.33	1.34 ± 1.35	1.36 ± 1.50	**p=0.0273**	p>0.9999	p=0.0664
**Number of GdE lesions†**	0.23 ± 0.53	0.00 ± 0.00	0.14 ± 0.47	p=0.1250	p=0.5000	p=0.7813
**Number of new T2w lesions†, §**	1.29 ± 0.47	0.14 ± 0.48	0.36 ± 1.50	**p=0.0005**	p>0.9999	**p=0.0001**
**Percentage of relapse-free patients¶**	59%	95%	95%	OR= 0.07 (95% IC: 0.01-0.46) **p=0.0040**	OR= 1.00 (95% IC: 0.05-19.82) p>0.9999	OR= 0.07 (95% IC: 0.01-0.46) **p=0.0040**
**Percentage of CDP-free patients¶**	–	100%	86%	–	OR= ∞ (95% IC: 0.91-∞) p=0.0728	–
**Percentage of patients with GdE lesions¶**	18%	0%	9%	OR= ∞ (95% IC: 0.95-∞) **p=0.0402**	OR= 0.00 (95% IC: 0.00-2.44) p=0.1760	OR= 2.44 (95% IC: 0.51-13.73) p=0.3218
**Percentage of patients with T2w lesions¶**	100%	10%	10%	OR= ∞ (95% IC: 22.55-∞) **p<0.0001**	OR= 1.05 (95% IC: 0.15-7.23) p=0.9610	OR= ∞ (95% IC: 23.59-∞) **p<0.0001**
**Percentage of NEDA-3 patients¶**	–	86%	68%	–	OR= 2.80 (95% IC: 0.66-11.06) p=0.1737	–

Clinical and magnetic resonance imaging (MRI) variables were measured in multiple sclerosis (MS) patients before starting dimethyl fumarate (DMF) treatment (baseline) and after 1 and 2 years of therapy.

†For numerical variables, p-values were calculated using the Wilcoxon signed rank test. Values are the mean +/- SD.

‡For baseline ARR, only the previous year was considered.

§Data not available for naïve MS patients at baseline, as most of them had a single MRI.

¶For percentages, odds ratio, confidence interval and p-values were calculated using the Chi-square test.

p<0.05 was considered statistically significant.

ARR, annualized relapse rate; EDSS, expanded disability status scale; GdE, gadolinium-enhanced T1 lesions; T2w, T2-weighted lesions; CDP, confirmed disease progression; NEDA, no evidence of disease activity.

Bold values are values statistically significant (*p<0.05).

In addition, the results shown in **Table 2** suggest that the clinical response to DMF is favorable at 1 year in most of patients, while at 2 years a higher percentage of patients begin to experience radiological activity or disease progression, indicating that DMF efficacy could be diminishing after the first year. Taking this into account, we considered more useful to predict the clinical response at 2 years, and classified patients as responders or non-responders to DMF using NEDA-3 at 2 years. As shown in **Table 1**, NEDA-3 and EDA-3 patients presented similar characteristics at baseline, except for a higher ARR in the EDA-3 subgroup.

### 3.2 Flow-cytometric characterization of monocyte and lymphocyte subpopulations in HD and MS patients at baseline and after 1 year of DMF treatment

A total of 24 subpopulations were found to be significantly different between HD and MS patients at baseline (**Supplementary Table 2**). Among them, MS patients presented a higher percentage of monocytes and interleukin 22 (IL-22) producing cells (p=0.0433 and p=0.0004, respectively). MS patients also presented a lower percentage of natural killer (NK) cells (p=0.0202) and, within them, the proportion of NK bright and NK dim was also altered (p=0.0069 for both). In addition, MS patients presented lower percentages of memory cells, such as effector memory CD4+ T cells (CD4 TEM) (p=0.0222), central memory CD4+ T cells (CD4 TCM) (p=0.0071), CD20+ memory B cells (MemB1) (p=0.0175), CD19+ memory B cells (MemB2) (p=0.0474) and class-switched memory B cells (CS MemB) (p=0.0034). In contrast, the percentages of CD20+ naïve B cells (NaïveB1) (p=0.0311), and CD19+ naïve B cells (NaïveB2) (p=0.0002) were increased. Interestingly, MS patients presented lower percentages of regulatory T cells (Treg) (p=0.0002) and regulatory B cells (Breg) (p=0.0465). Lower proportions of transitional B cells (TransitB) (p=0.0137), interferon gamma (IFNγ) producing cells (p=0.0071) and IL-4 producing cells (p=0.0030) were also found in MS patients at baseline.

Furthermore, we investigated whether the differences found between MS patients and HD could be due to the effect of previous immunomodulatory therapies used in MS, by comparing patients who had received prior treatment with naïve patients (**Supplementary Table 3**). However, few differences were found between these subgroups and among the subpopulations that differed between MS and HD, only NK bright, NK dim and IL-4-producing T cells differed between naïve and previously treated patients.

Regarding the effects of 1 year of DMF treatment on the monocyte and lymphocyte subpopulations analyzed, we observed changes in at least 30 different subsets (**Supplementary Table 4**). DMF produced a decrease in cytotoxic (p=0.0008), NKT (p=0.0005) and B cells (p=0.0229), while helper T cells (p=0.0066) and NKs (p=0.0462) were found to be increased. DMF also reduced the percentages of memory TEM and TCM cells both in the CD4+ (p=0.0002 and p=0.0017, respectively) and CD8+ (p<0.0001 and p=0.0001, respectively) subsets. Accordingly, an increase in naïve T cells was also found, although only in the CD4+ subset (p=0.0002). A similar effect was observed on B lymphocytes with a reduction in memory B cells MemB1 (p=0.0275) and MemB2 (p=0.0215), including CS MemB (p=0.0136) and non-class switched memory B cells (NoCS MemB) (p=0.0484), and in the CD11b+ B lymphocytes (p=0.0266). Regulatory T (p=0.0351) and B cells (p=0.0362) were also decreased after 1 year. DMF also decreased the cytokine-producing T cells IFNγ+ (p<0.0001), IL-17+ (p=0.0051), IL-2+ (p=0.0037) and IL-4+ (p=0.0470).

### 3.3 Differences in monocyte and lymphocyte subpopulations between responders and non-responders to DMF

**Figure 1:**
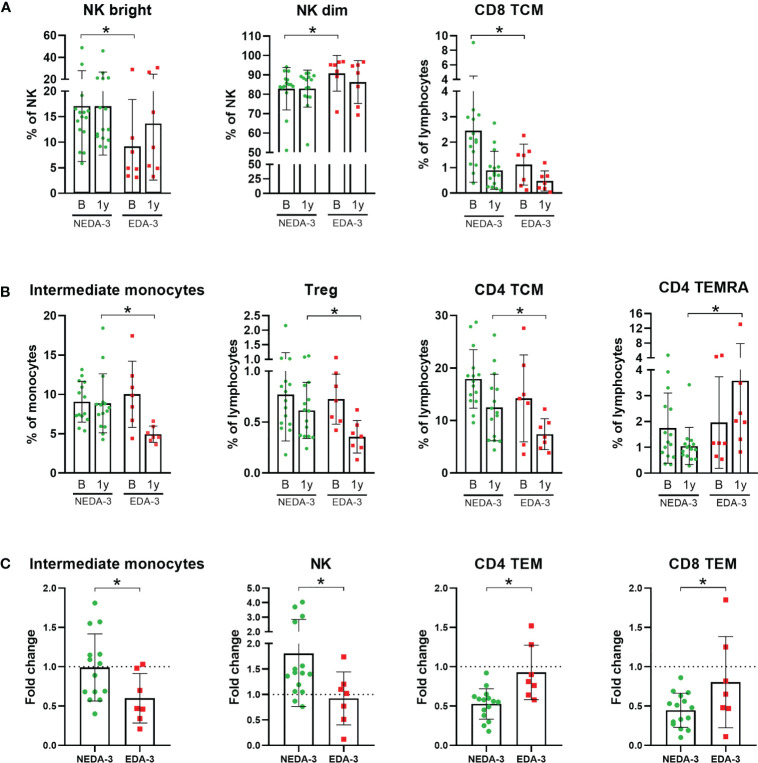
Monocyte and lymphocyte subpopulations with significant differences between responder and non-responder patients to dimethyl fumarate. **(A)** Subpopulations with significant differences at baseline: Natural killer cells (NK) bright, NK dim and CD8+ central memory T cells (TCM). **(B)** Subpopulations with significant differences at 1 year: Intermediate monocytes, regulatory T cells (Treg), CD4 TCM and CD4+ effector memory cells re-expressing CD45RA T-cells (TEMRA). **(C)** Subpopulations which varied differentially across time between responders and non-responders: Intermediate monocytes, NK, CD4+ effector memory T cells (TEM) and CD8 TEM. Fold change was calculated as 1 year/baseline. The Mann-Whitney test was used to compare differences between NEDA-3 (no evidence of disease activity-3) and EDA-3 (evidence of disease activity-3) patients. *p<0.05 was considered statistically significant.

As shown in **Figure 1A**; **Supplementary Table 5**, only 3 subpopulations were found to be different between responders and non-responders to DMF at baseline. Interestingly, among these 3 subpopulations were the NK bright and NK dim subsets, which also predicted treatment response to fingolimod (17). Patients who achieved NEDA-3 presented a higher percentage of NK bright cells and, consequently, a lower proportion of NK dim cells (p=0.0164 for both). Patients who achieved NEDA-3 also presented a higher percentage of CD8 TCM (p=0.0387) compared to EDA-3 patients. However, none of these differences were maintained after 1 year of DMF treatment.

At 1 year, 4 different subpopulations presented distinctive percentages between responders and non-responders (**Figure 1B**). NEDA-3 patients had higher proportions of Treg (p=0.0480), CD4 TCM (p=0.0465) and intermediate monocytes (p=0.0021), and lower proportions of CD4 effector memory cells re-expressing CD45RA T-cells (TEMRA) (p=0.0064). In addition, we also considered the changes in the subpopulations between NEDA-3 and EDA-3 patients, comparing the 1-year values with baseline (**Figure 1C**). The population of intermediate monocytes decreased after 1 year in both responders and non-responders; however, NEDA-3 patients were more resistant to this decrease (p=0.0461). NK cells also varied differentially during DMF treatment, with NEDA-3 patients experiencing an increase and EDA-3 a decrease (p=0.0387). Moreover, NEDA-3 patients also experienced a decrease in the CD4 TEM subset, while EDA-3 remained unaltered (p=0.0029). CD8 TEM cells also decreased with DMF in both groups, being the EDA-3 group more resistant to this reduction (p=0.0162).

### 3.4 Effects of DMF treatment on gene expression

**Figure 2:**
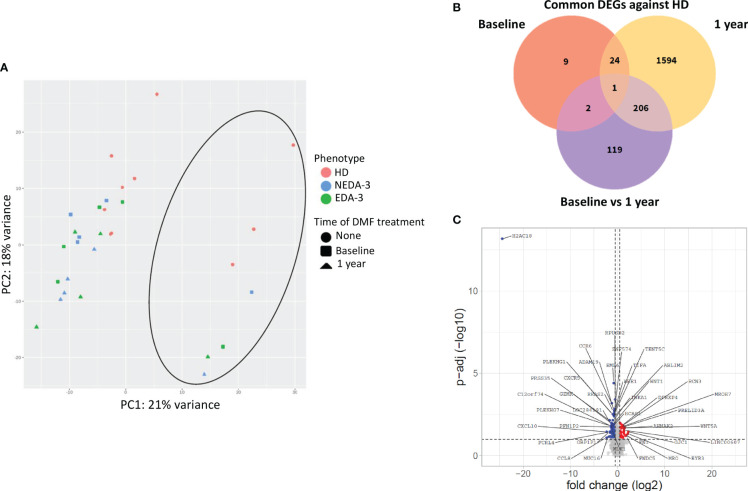
Effects of DMF treatment on gene expression. **(A)** Principal component analysis of the gene expression profile of HD and MS patients at baseline and after 1 year of DMF treatment. The circle indicates the cluster formed by male samples. **(B)** Venn diagram showing the overlap of the differentially expressed genes (DEGs) between HD and MS at baseline and after 1 year of DMF treatment. **(C)** Volcano plot showing DEGs in MS patients between baseline and 1 year of DMF treatment. p-adj<0.05 was used as the threshold to judge the significance of the difference in gene expression. The genes with greater fold changes and statical significance are indicated with their names. Red plots represent upregulated genes; blue plots represent downregulated genes; grey plots represent genes with no significant differences.

**Figure 2A** shows a principal component analysis of the gene expression profiles in HD and MS patients. HD tend to situate in a differentiated region of space compared to MS patients, although this separation was not completely defined. Samples from MS patients grouped all together independently of the time of DMF treatment, indicating a mild transcriptional effect of DMF on PBMCs. Within the group of MS patients, no aggregation was obtained either between responders and non-responders neither at baseline nor at 1 year, suggesting that there is not a clearly differentiated transcriptomic profile between NEDA-3 and EDA-3 patients. Interestingly, a cluster of samples (indicated with a circle) separated themselves from the rest. These samples corresponded to male individuals, suggesting that female and male samples separate into two clearly discernible groups due to differential transcriptomic profiles. This differentiation by sex was clear in both HD and in MS. This led us to investigate the differences between female and male patients in the subpopulations obtained by flow cytometry (**Supplementary Table 6**). Although not many subpopulations were found dysregulated between women and men, we found differences in highly represented subpopulations, with women presenting higher percentages of T lymphocytes (p=0.0063) and cytotoxic T cells (p=0.0150) and a lower proportion of B lymphocytes (p=0.0086). Women also had higher numbers of non-classical monocytes (p=0.0137) and plasmatic cells (p=0.0493) and lower numbers of IL-4-producing T cells (p=0.0294).

When analyzing the differentially expressed genes (DEGs) between HD and MS patients at baseline, only 36 genes were dysregulated (**Figure 2B**). After 1 year of DMF treatment, the number of DEGs between HD and MS increased to 1825. Between baseline and 1 year of DMF treatment, the number of DEGs in MS patients was 328 (264 downregulated 64 and upregulated). As shown in **Figure 2C**, DMF produced a downregulation of pro-inflammatory chemokine genes such as *CXCL10*, *CCR6*, *CXCR6*, *CCR9*, *CXCR5* and *CXCR3*. DMF also downregulated the pro-inflammatory genes *CD8A*, *CD8B*, *IL2RG*, *IL12A*, *CD70*, *CD79A*, *CD79B* and *FCRL4*. Other downregulated transcripts included the immune checkpoint *LAG3* and activators of the NF-kB pathway such as *TNFRSF13B*, *TIFA*, *TRAF4*, *NUAK2*, *PRKCZ* and *TRIM13*.

### 3.5 Gene expression in responder and non-responder patients to DMF

**Figure 3:**
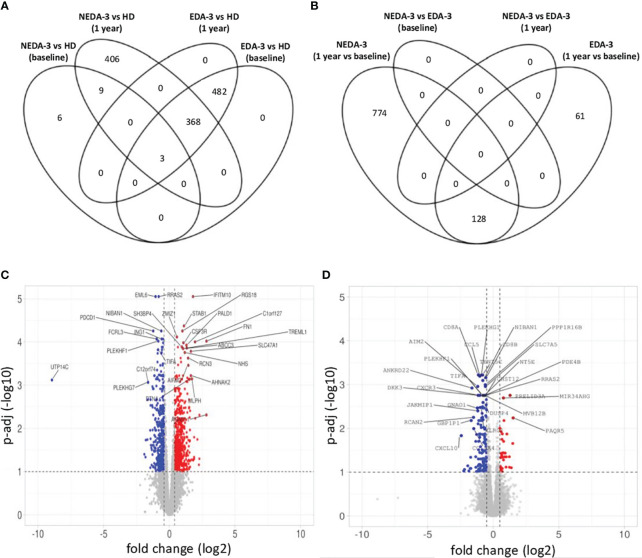
Gene expression in responder and non-responder patients to DMF. **(A)** Venn diagram showing the overlap of the differentially expressed genes (DEGs) between HD and responders and non-responders to DMF at baseline and after 1 year of treatment. **(B)** Venn diagram showing the overlap of the DEGs between responders and non-responders to DMF at baseline and after 1 year of treatment. **(C)** Volcano plot of the DEGs in responder patients between baseline and 1 year of DMF treatment. **(D)** Volcano plot of the DEGs in non-responder patients between baseline and 1 year of DMF treatment. C-D. p-adj<0.05 was used as the threshold to judge the significance of the difference in gene expression. The genes with greater fold changes and statical significance are indicated with their names. Red plots represent upregulated genes; blue plots represent downregulated genes; grey plots represent genes with no significant difference. The genes with greater fold changes and statical significance are indicated (adjusted p-value<0.05).

When analyzing HD against MS patients considering their response to treatment, we found that responders had a lower number of DEGs (18 DEGs) at baseline compared to non-responders (853 DEGs) (**Figure 3A**). At 1 year, the number of DEGs in responders against HD increased to 786, while in non-responders the number of DEGs remained stable (**Figure 3A**). In the paired analysis, no DEGs were found between responder and non-responder patients at baseline (**Figure 3B**). However, during DMF treatment a differential transcriptomic response was observed, with responders having a higher number of DEGs (902 genes) compared to non-responders (189 genes). Of this DEGs, 128 were common in both groups. Responder patients presented downregulation of genes related to pro-inflammatory cytokines and their receptors (*IL32*, *IL2R*, *IL18R1*, *IL15RA*, *IFNLR1*, *IL12RB1*, *IL12RB2*, *IL3RA*, *CD70*, *STAT1*), absent in the non-responder group (**Figure 3C**). Responders also presented a higher number of DEGs related to chemokines (*CXCR6*, *CCR9*, *CXCL11*, *CXCL2*, *CXCL3*) compared to non-responders. In addition, responder patients exhibited several DEGs involved in the regulation of T-cell functions (*VSIG4*, *LOXL3*, *CD80*, *PTPN7*, *NFATC2*, *CD2*) and in the NF-kB pathway (*NLRP12*, *IFIT5*, *CARD11*, *PRKCZ*, *PRKCQ*, *RELT*, *RNF25*).

Non-responders (**Figure 3D**) displayed downregulation of important pro-inflammatory genes such as *CD8B*, *GZMK*, *GZMH*, *TNF* and *IFNG*. However, a downregulation of genes implicated in the control of immune responses, especially in T lymphocytes, was also observed, including *KLRG1*, *TIGIT*, *CST7*, *NT5E*, *SLA2*, *IRF1*, *AP1S3*, *RCAN2* and *GPR18*. This could be indicating a deficient regulation of inflammation in non-responder patients.

## 4 Discussion

In this article, we describe the immunophenotype and transcriptomic profile of PBMCs from MS patients treated with DMF. At least 30 flow cytometry subpopulations changed after 12 months of DMF treatment, although only a mild transcriptomic effect was observed, with 328 DEGs compared to baseline. In addition, clinical response to DMF could be associated with several monocyte and lymphocyte subpopulations, and a distinguishable transcriptomic response was observed between responders and non-responders to the drug.

The immunomodulatory effect of DMF has been explored at different time points in several studies using small cohorts of MS patients. The overall changes induced by the drug include a decrease in memory T and B cells and a subsequent increase in naïve cells, as well as a shift in cytokine production towards an anti-inflammatory profile. In line with previous studies on the T cell subsets, we have observed a decrease in memory TEM and TCM cells (both CD4+ and CD8+) and an increase in naïve T cells (CD4+ only) at 12 months (10–12). Moreover, we have found a differential regulation in the CD4+ and CD8+ TEM subpopulations according to the clinical response, with responders presenting a greater decrease compared to non-responders. A similar association to that of TEM cells has been described in TCM lymphocytes, which decreased only in the responder group (11). In our cohort, TCM cells did not vary differentially depending on the clinical response, as they decreased with DMF treatment in both responder and non-responder patients. However, responders had higher percentages of CD8+ TCM cells at baseline and CD4+ TCM cells at 1 year. Another population of T lymphocytes associated with clinical response to DMF was CD4+ TEMRA, with responders presenting lower percentages at 1 year. These results suggest that memory T lymphocytes could have the potential to be used as biomarkers of clinical response to DMF.

Regarding the cytokine profile, DMF produced a strong decrease in the Th1 response, halving the percentages of IFNγ and IL-2 producing T cells in both the CD4+ and CD8+ subsets, in line with previous studies (10, 12, 23–26). Although a greater decrease in IFNγ-producing CD4+ T cells has been associated with responder patients to DMF (11), in our study we did not find any association with clinical outcomes with either IFNγ or IL-2. In our cohort we have also observed a decline in the Th17 response, with a reduction in the percentages of IL-17A producing total T lymphocytes and within them in the CD4+ subset but not in the CD8+. The effects produced by DMF on the Th17 response are less consistent between studies, as some reported the same decrease (10, 23, 27), while others have described no changes (12, 24, 25). In addition, we did not find any association between IL-17+ CD8 T cells and clinical response as previously described (16), which could be due to the extremely low representation of these cells. As for the Th2 response, we have observed a slight decline in the percentages of IL-4 producing T cells in both the CD4+ and CD8+ subpopulations. The levels of IL-4 have been found to be both increased (10, 27) or unchanged (12, 23–26) during treatment with DMF, suggesting that the precise effect of DMF on IL-4 remains to be elucidated.

With regard to regulatory T cells, we have found that responder patients presented higher percentages of these cells at 1 year compared to non-responders. Most of the studies have found that the proportions of regulatory T cells remain unaltered (9–11, 23, 27, 28) or increase (12, 24) during DMF treatment. In our study we observed a slight decline in the total cohort. However, although this difference was not statistically significant, the decrease in regulatory T cells was 50% in the non-responder group while only 20% in responders. This could be indicating that the decline observed during DMF treatment could be influenced by a differential response between these subgroups of patients and that regulatory T cells could be more impaired in non-responders. In addition, DMF has been reported to induce methylation changes involved in the regulation and function of regulatory T cells (15), suggesting that DMF influences these cells regardless of the absence of changes in their total counts.

In the B cell compartment, we have found a decrease in CD27+ memory B cells and, within them, in both subpopulations of class-switched and non-classed switched lymphocytes, in accordance with previous studies (13, 23, 25, 27, 29, 30). Although the percentages of naïve B cells have been found to be proportionally increased during DMF treatment (23, 25, 29, 30), in our study we observed an increase but it was not statistically significant (p=0.0587 for NaïveB1 and p=0.1621 for NaïveB2). On the other hand, the percentages of plasmablasts and plasmatic cells remained unaltered after 12 months of DMF treatment as previously described (11, 29, 30). We did not find any association between B lymphocytes and clinical response to DMF and, as far as we know, only one study has related a decrease in TNFα producing B lymphocytes and optimal response to DMF (11), suggesting that B lymphocyte subpopulations are not good candidates as biomarkers of response to treatment with DMF.

Regarding NK cells, we have found that, although there were no baseline differences in the percentages of the total NK population, within them, the responder patients presented higher percentages of CD56bright NK cells and, consequently, lower percentages of CD56dim cells compared to non-responders. Interestingly, in a previous study from our group we described that the subpopulation of CD56bright NK cells was also higher at baseline in responder patients to fingolimod (17). Expansion of CD56bright NK cells has also been found in responder patients after treatment with daclizumab (31), interferon beta (32) and DMF (11, 33). In our cohort, we were unable to observe an expansion of CD56bright NK cells after DMF treatment, which could be due to the already existing baseline differences between responders and non-responders. However, we found a differential regulation in the NK total subset, with responders presenting an increase after 1 year of DMF treatment while non-responders showed a decrease.

The effect of DMF on the innate immune system has been less examined. Although most of the studies have found no changes in the absolute monocyte count (9, 10, 23, 27), only one study has focused on the classical and non-classical monocyte subpopulations (27), describing no changes and a decline in their percentages respectively. In our cohort of patients, the percentages of total monocytes and of the 3 subpopulations remained unchanged after 1 year of DMF therapy. However, we have found that the subset of intermediate monocytes changed differentially between NEDA-3 and EDA-3 patients, with the NEDA-3 subgroup being more resistant to a decrease. Interestingly, a previous study already reported that the presentation of increased monocyte counts and monocytic ROS following DMF treatment distinguished responder from non-responder MS patients (15).

In this study we have also explored the transcriptomic effect produced by DMF and tested the hypothesis that responder and non-responder patients would exhibit a differential gene expression regulation. Using RNA-seq, we were able to identify only 328 DEGs after 1 year of treatment, indicating that DMF produces a mild transcriptional effect. The overall response was a downregulation of pro-inflammatory genes, mainly chemokines, and the inhibition of the NF-kB pathway. A previous transcriptomic study in RRMS patients found a similar number of DEGs (478) at 6 weeks of DMF treatment, which were enriched for transcripts related to the nuclear factor E2-related factor 2 (Nrf2) and NF-kB pathways (34). Both signalling pathways have been proposed as possible mechanisms of action of DMF (35). The NF-kB transcription factor is considered a master regulator of the immune system due to its role on the regulation of pro-inflammatory genes and on the survival, activation and differentiation of lymphocytes (36). The inhibition of the NF-kB pathway would explain many of the immunomodulatory effects observed in this study such as the expression changes in chemokines and cytokines. In fact, DMF-induced inhibition of NF-kB has been directly linked with reduced production of inflammatory cytokines and altered function of antigen-presenting cells (13, 37, 38). On the other hand, the activation of the Nrf2 pathway induced by DMF has also been demonstrated in numerous studies (35). However, the role of Nrf2 in controlling inflammation has been compromised due to the finding of DMF-induced anti-inflammatory activity via Nrf2- independent mechanisms (38). This was highlighted in the mouse model of MS, experimental autoimmune encephalomyelitis (EAE), where Nrf2 (-/-) mice were equally protected from the disease as wild-type mice (39). Our transcriptomic results reinforce the idea that the suppression of the NF-kB pathway is mainly responsible for the immunomodulatory effects achieved by DMF. The lack of DEGs related to the Nrf2 pathway in our study at 12 months, in contrast with the enrichment found at 6 weeks (34), could be indicating that Nrf2 activation is associated with early short-term transcriptomic changes that are lost at a later stage of therapy.

With respect to clinical response, the small sample size of our cohort limited the identification of DEGs at baseline that could be useful for predicting treatment response to DMF. However, during DMF treatment a differential gene expression regulation was observed with responders presenting a higher number of DEGs compared to non-responders. Similar results had been found at 6 weeks of DMF treatment, where responder patients presented a higher number of DEGs (34). In responder patients we observed a modulation of pro-inflammatory genes related to cytokines and chemokines that was absent in the non-responder group. In addition, suppression of the NF-kB pathway was also associated with responder patients, suggesting that the modulation of this pathway is deficient in non-responders and could be related to a suboptimal response to DMF.

Finally, we also compared the transcriptomic profile of RRMS patients at baseline and at 1 year of DMF treatment with that of HD. Responders at baseline were more similar to HD than non-responders, suggesting that responders could have a less dysregulated immune system that would be easier to modulate by DMTs including DMF. Non-responders presented the same differences at baseline and at 1 year compared to HD, indicating the lack of efficacy of DMF in modulating gene expression in this group of patients.

## 5 Conclusions

Responder patients to DMF show a differential regulation in the intermediate monocytes, NK cells and TEM cells (both CD4+ and CD8+) subpopulations, also supported by a differential transcriptomic response that affects primarily the NF-kB pathway. This differential regulation should be further studied for the validation of biomarkers of response to DMF.

## Data availability statement

The datasets used and/or analyzed during the current study are available from the corresponding author on reasonable request. The data presented in the study are deposited in the NCBI's Gene Expression Omnibus repository, and are accessible through GEO Series accession number GSE235357 (https://www.ncbi.nlm.nih.gov/geo/query/acc.cgi?acc=GSE235357).

## Ethics statement

The studies involving human participants were reviewed and approved by University Hospital Puerta de Hierro Ethics Committee. The patients/participants provided their written informed consent to participate in this study.

## Author contributions

AS-S performed the experiments, analyzed, and interpreted the data, and wrote the manuscript. SG-M and FA-S performed bioinformatic analyses. JS-M, OR-D, and RG-H recruited and treated patients. IM-T conceived and designed the study. VE analyzed the data. AG-G and ER, methodological support. BB-A, acquisition and recording of MRI. AG-M and AS-L conceived and designed the study and interpreted the data. All the authors have read and contributed to the final version of the manuscript and approve the submission for publication.
