# Supplementary material for: Dimethyl fumarate-related immune and transcriptional signature is associated with clinical response in multiple sclerosis-treated patients
Source: Front Immunol. 2023 Jul 7;14:1209923. doi: 10.3389/fimmu.2023.1209923 (PMC10360655; doi:10.3389/fimmu.2023.1209923)
Supplement: Supplementary file 1 [file DataSheet_1.pdf]

Supplementary figure S1. Gating strategy used in flow cytometry

Representative data from a patient are shown. Lymphocytes and monocytes were gated on a forward scatter (FSC)/side scatter (SSC) plot. Live cells were gated by 4′,6-diamidine-2′-phenylindole dihydrochloride (DAPI) in S1.1-S1.5 and by the fluorescent reactive dye from the LIVE/DEAD™ Fixable Violet Dead Cell Stain Kit (Thermo Fisher Scientific) in S1.6-S1.8.

S1.1

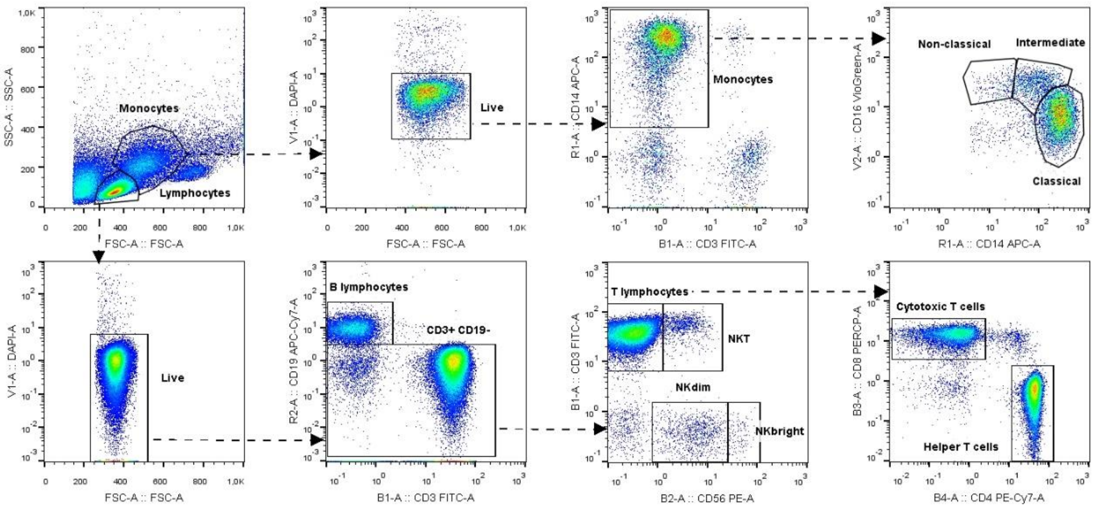

S1.1 Gating strategy in panel 1: Live monocytes were gated to determine CD3-CD14+ (monocytes). Monocytes were further gated to determine CD14highCD16- (classical monocytes), CD14highCD16+ (intermediate monocytes) and CD14+CD16high (non-classical monocytes). Live lymphocytes were gated to determine CD19+CD3- (B lymphocytes) and CD3+CD19-. CD3+CD19- were gated to determine CD3+CD56- (T lymphocytes), CD3+CD56+ (natural killer T cells, NKT), CD3-CD56dim (natural killer cells, NK) (NKdim) and CD3-CD56bright (NKbright). T lymphocytes were further gated into CD4+CD8- (helper T cells) and CD4-CD8+ (cytotoxic T cells).

S1.2

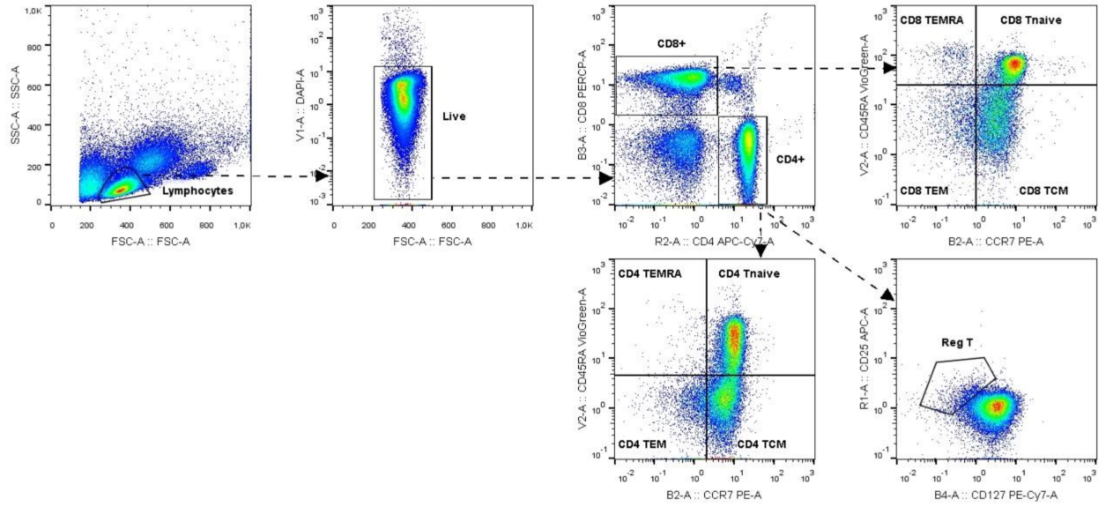

S1.2 Gating strategy in panel 2: Live lymphocytes were gated to determine CD4+CD8- (helper T cells) and CD4-CD8+ (cytotoxic T cells). Helper T cells were gated into CCR7-CD45RA- (effector memory T cells, CD4 TEM), CCR7+CD45RA- (central memory T cells, CD4 TCM), CCR7-CD45RA+ (effector memory RA T cells, CD4 TEMRA), and CCR7+CD45RA+ (naïve T cells, CD4 Tnaïve). Helper T cells were also gated in CD127downCD25high (regulatory T cells, Treg). Cytotoxic T cells were gated into CCR7-CD45RA- (CD8 TEM), CCR7+CD45RA- (CD8 TCM), CCR7-CD45RA+ (CD8 TEMRA) and CCR7+CD45RA+ (CD8 Tnaïve).

S1.3

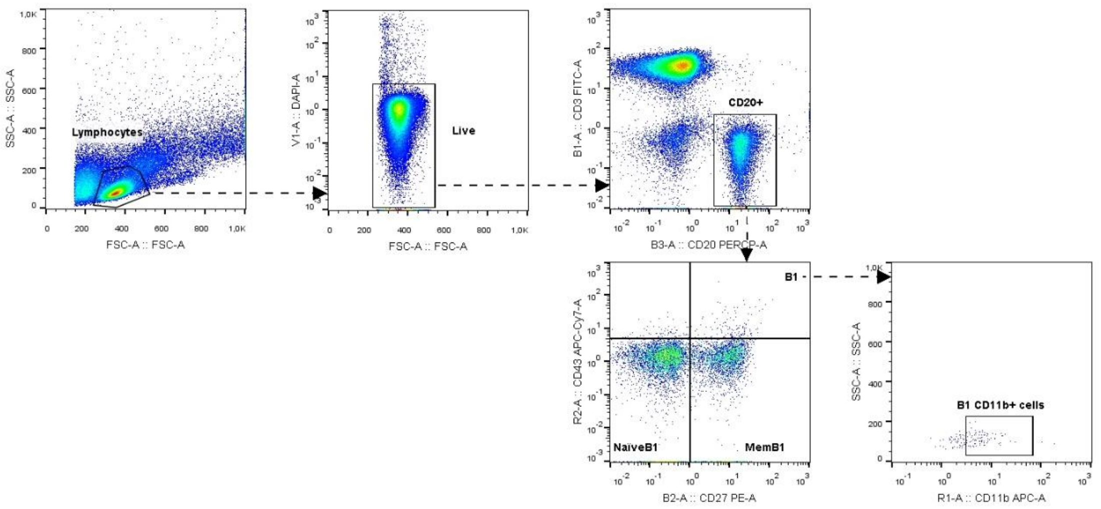

S1.3 Gating strategy in panel 3: Live lymphocytes were gated to determine CD3-CD20+ cells. CD3-CD20+ were gated into CD27+CD43- (CD20 memory B cells, MemB1), CD27-CD43- (CD20 Naïve B cells, NaïveB1), and CD27+CD43+ (B1 cells). B1 cells were further gated into B1 CD11b+ cells on SSC.

S1.4

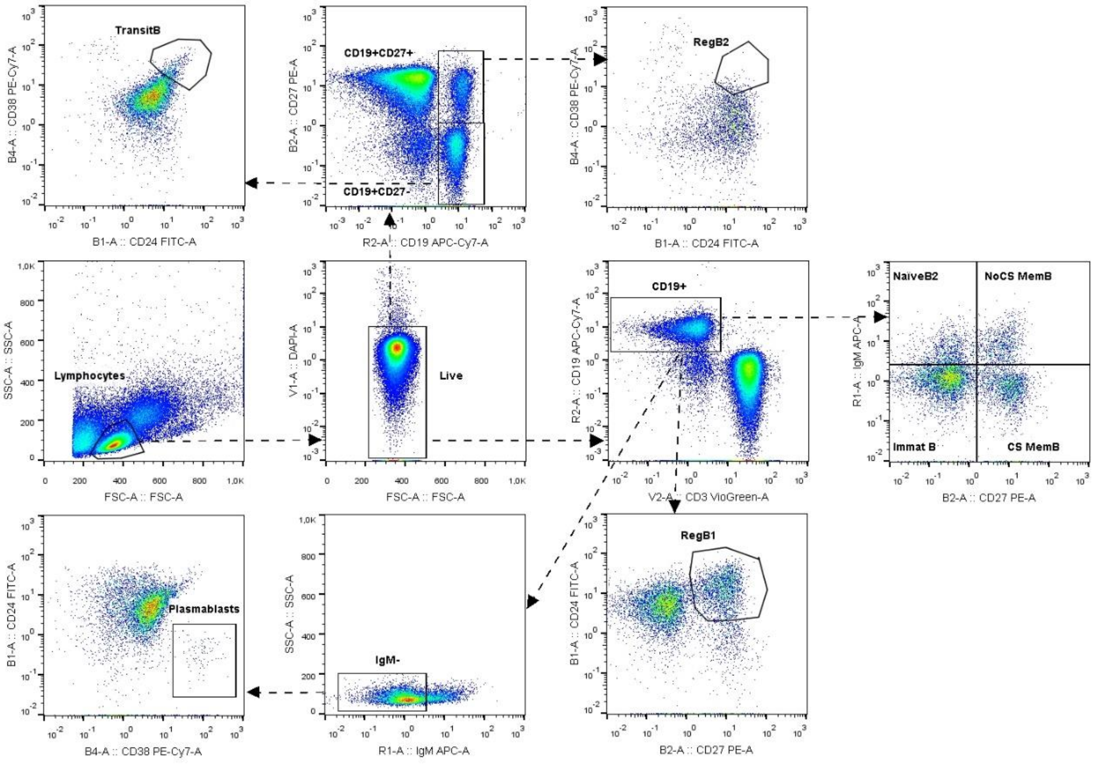

S1.4 Gating strategy in panel 4: Live lymphocytes were gated to determine CD19+CD3- cells. CD19+ were gated into IgM-CD27- (immature B cells, Immat B), IgM + CD27- (CD19 Naïve B cells, NaïveB2), IgM-CD27+, (class switched memory B cells, CS MemB) and IgM+CD27+ (non-classed switched memory B cells, NoCS MemB). CD19+ were also gated into IgM-, and IgM- into CD24-CD38high (plasmablasts). CD19+ were also gated into CD27+CD24high (regulatory B cells, RegB1). Live lymphocytes were also gated into CD19+CD27- and CD19+CD27+. CD19+CD27+ were gated into CD24highCD38high (regulatory B cells, RegB2). CD19+CD27- were gated into CD24highCD38high (transitional B cells, TransitB).

S1.5

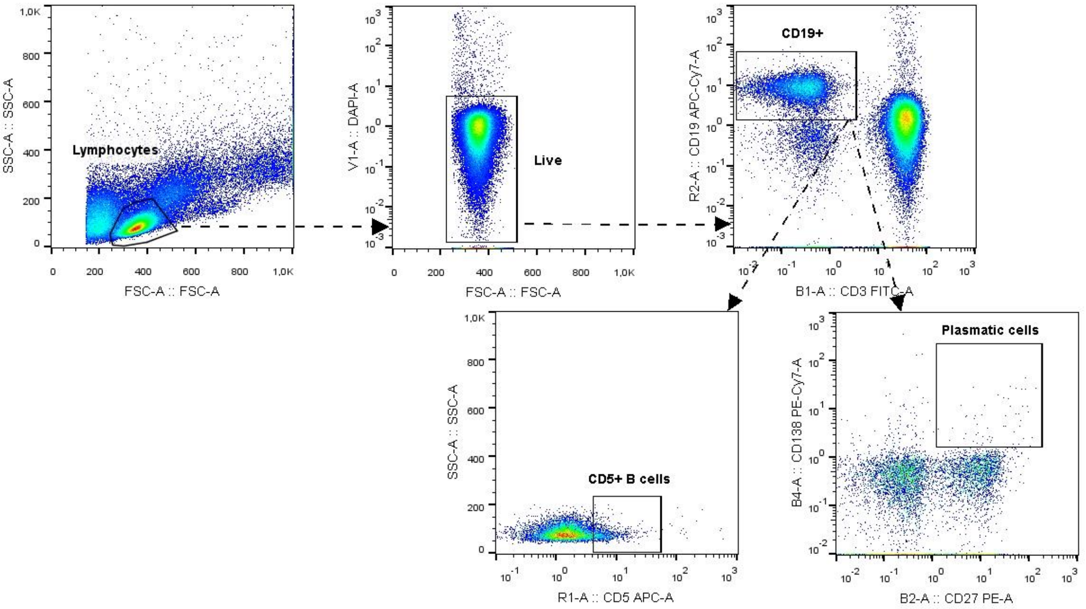

S1.5 Gating strategy in panel 5: Live lymphocytes were gated to evaluate CD19+CD3– cells. CD19+CD3- cells were gated into CD138+CD27+ (plasmatic cells) and into CD5+ B cells on SSC.

S1.6

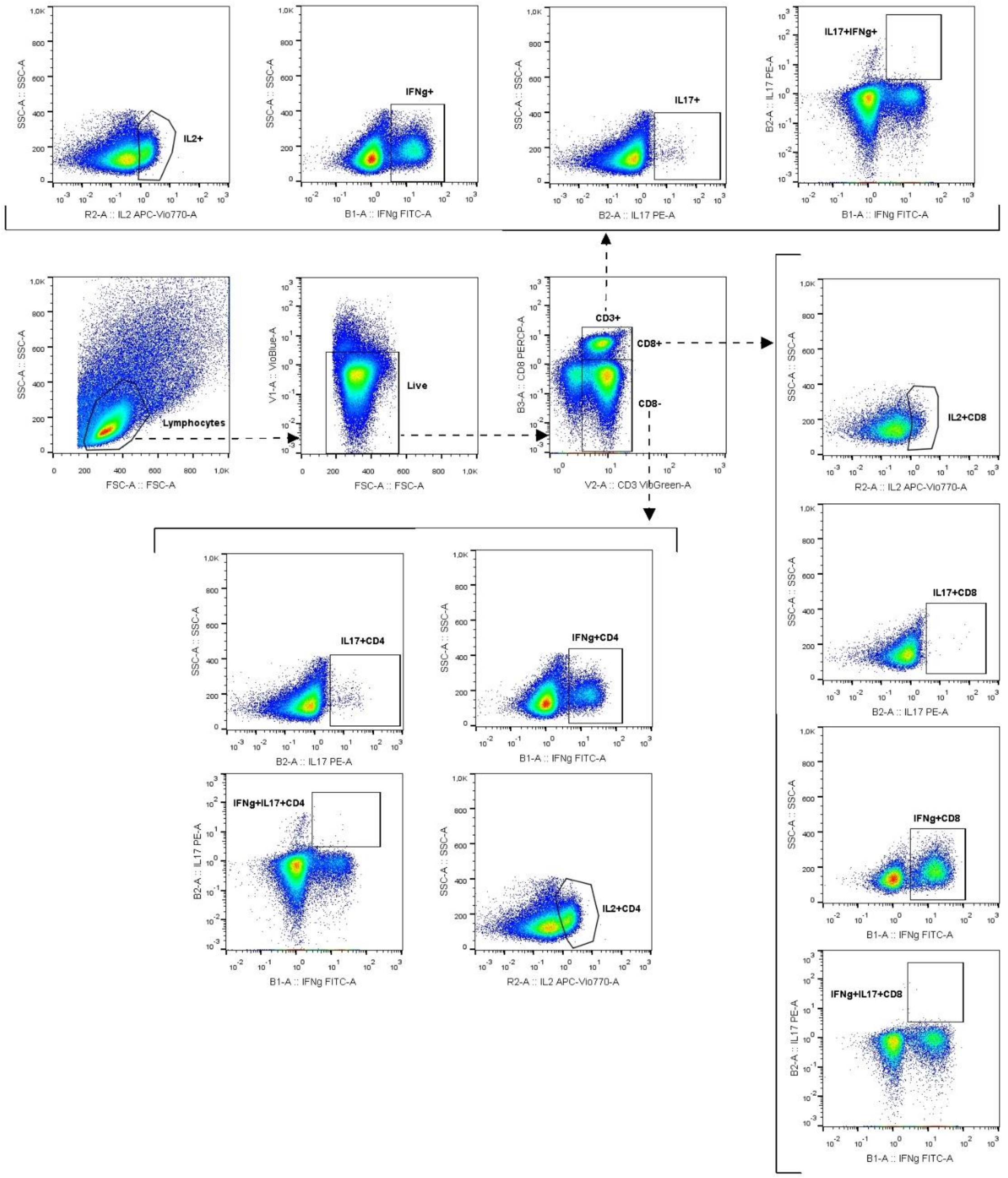

S1.6 Gating strategy in panel 6: Live lymphocytes were gated into CD3+ (T lymphocytes), CD3+CD8- (helper T cells) and CD3+CD8+ (cytotoxic T cells). For each subpopulation, the percentage of IL-17, IL-2 and IFNγ producing cells was determined on a SSC plot. The percentage of IL-17+IFNγ+ producing cells was also determined for the 3 subpopulations of T lymphocytes.

S1.7

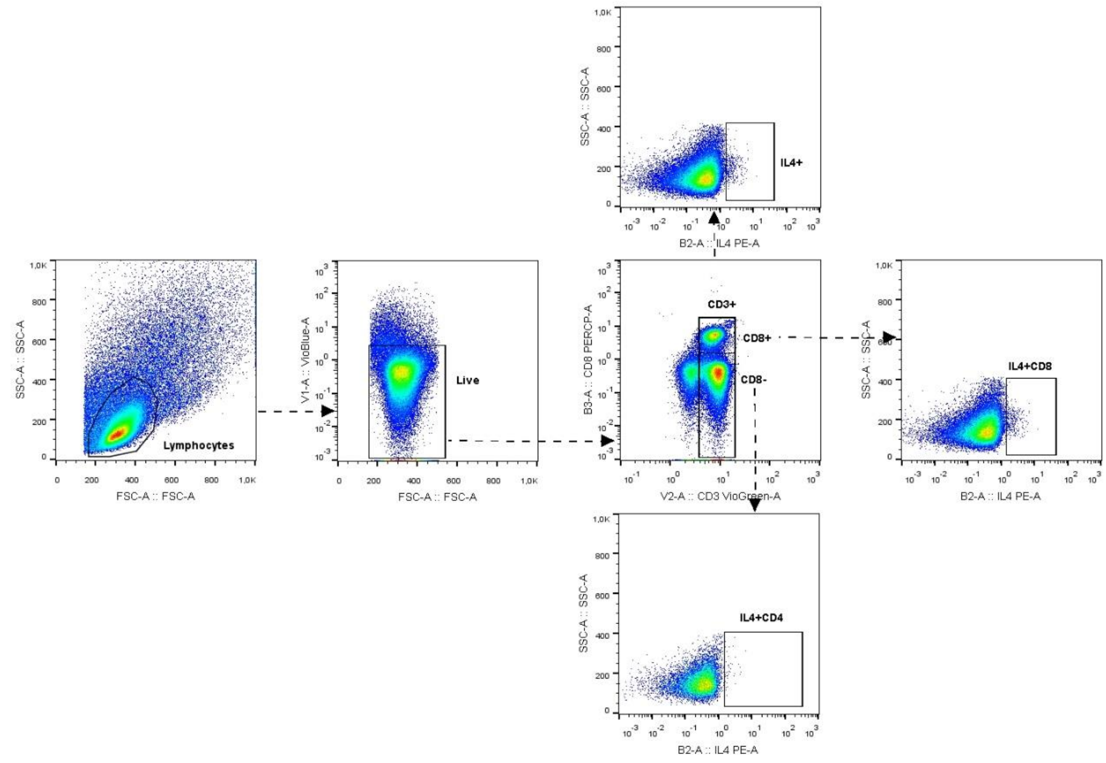

S1.7 Gating strategy in panel 7: Live lymphocytes were gated into CD3+ (T lymphocytes), CD3+CD8- (helper T cells) and CD3+CD8+ (cytotoxic T cells). For each subpopulation, the percentage of IL-4 producing cells was determined on a SSC plot.

S1.8

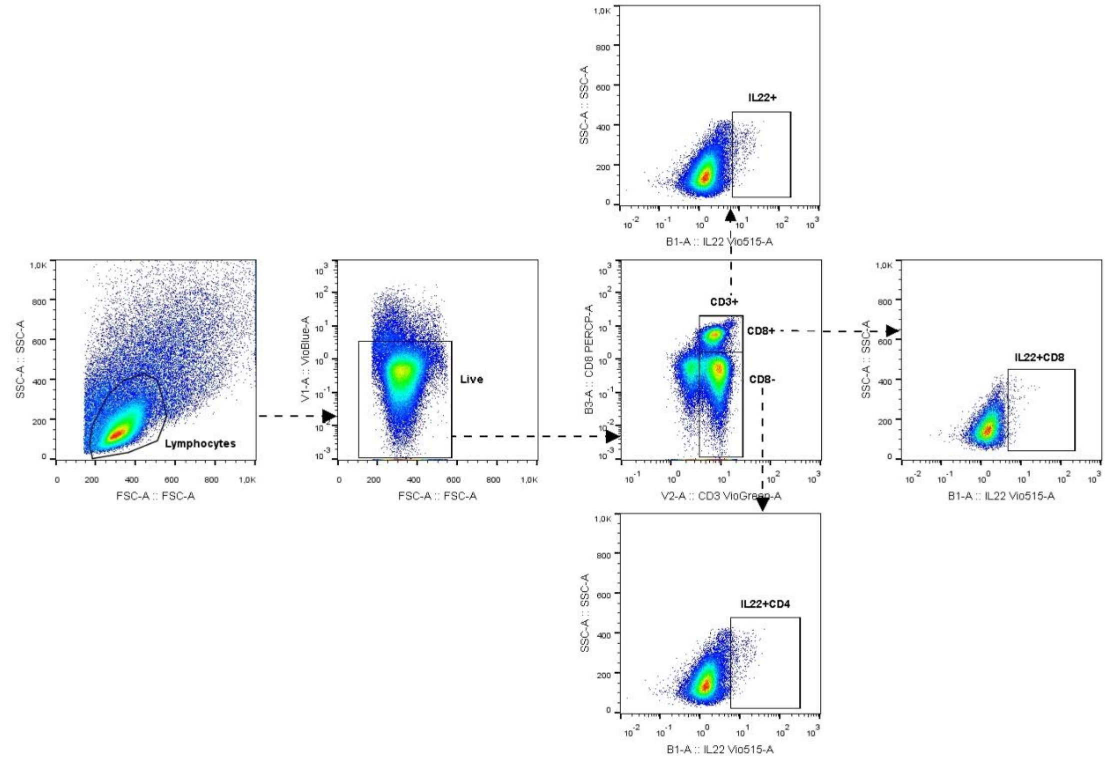

S1.8 Gating strategy in panel 8: Live lymphocytes were gated into CD3+ (T lymphocytes), CD3+CD8- (helper T cells) and CD3+CD8+ (cytotoxic T cells). For each subpopulation, the percentage of IL-22-producing cells was determined on a SSC plot.
