# Supplementary material for: Dimethyl fumarate-related immune and transcriptional signature is associated with clinical response in multiple sclerosis-treated patients
Source: Front Immunol. 2023 Jul 7;14:1209923. doi: 10.3389/fimmu.2023.1209923 (PMC10360655; doi:10.3389/fimmu.2023.1209923)
Supplement: Supplementary file 2 [file DataSheet_2.pdf]

Supplementary Table 1. Monocyte and lymphocyte subpopulations analysed by flow cytometry

PANEL	ANTIBODIES	SUBPOPULATIONS ANALYSED
**1**	anti-CD16 VioGreen<br>anti-CD3 FITC<br>anti-CD56 PE<br>anti-CD8 PerCP<br>anti-CD4 PE-Vio770<br>anti-CD14 APC<br>anti-CD19 APC-Vio770	**Monocytes:** CD3-CD14+<br>- **Classical:** CD3-CD14^{high}CD16-<br>- **Intermediate:** CD3-CD14^{high}CD16+<br>- **Non-classical:** CD3-CD14+CD16^{high}<br>**T lymphocytes:** CD3+CD19-CD56-<br>- **Helper T cells:** CD3+CD19-CD56-CD4+<br>- **Cytotoxic T cells:** CD3+CD19-CD56-CD8+<br>**B lymphocytes:** CD3-CD19+<br>**Natural Killer T cells (NKT):** CD3+CD56+CD16-<br>**Natural Killer cells (NK):** CD3-CD19-CD56+<br>- **NKbright:** CD3-CD19-CD56^{bright}<br>- **NKdim:** CD3-CD19-CD56^{dim}
**2**	anti-CD45RA VioGreen<br>anti-CCR7 PE<br>anti-CD8 PerCP<br>anti-CD127 PE-Vio770<br>anti-CD25 APC<br>anti-CD4 APC-Vio770	**Naïve T cells (Tnaïve):**<br>- **CD4 Tnaïve:** CD4+CD8-CCR7+CD45RA+<br>- **CD8 Tnaïve:** CD8+CD4-CCR7+CD45RA+<br>**Central memory T cells (TCM):**<br>- **CD4 TCM:** CD4+CD8-CCR7+CD45RA-<br>- **CD8 TCM:** CD8+CD4-CCR7+CD45RA-<br>**Effector memory T cells (TEM):**<br>- **CD4 TEM:** CD4+CD8-CCR7-CD45RA-<br>- **CD8 TEM:** CD8+CD4-CCR7-CD45RA-<br>**Effector memory re-expressing CD45RA T cells (TEMRA):**<br>- **CD4 TEMRA:** CD4+CD8-CCR7-CD45RA+<br>- **CD8 TEMRA:** CD8+CD4-CCR7-CD45RA+<br>**Regulatory T cells (RegT):** CD4+CD8-CD25^{high}CD127^{down}
**3**	anti-CD3 FITC<br>anti-CD27 PE<br>anti-CD20 PerCP<br>anti-CD11b APC<br>anti-CD43 APC-Vio770	**CD20 Naïve B cells (NaïveB1):** CD3-CD20+CD27-CD43-<br>**CD20 Memory B cells (MemB1):** CD3-CD20+CD27+CD43-<br>**B1 Cells (B1):** CD3-CD20+CD27+CD43+<br>**B1 CD11b+ cells:** CD3-CD20+CD27+CD43+CD11b+
**4**	anti-CD3 VioGreen<br>anti-CD24 FITC<br>anti-CD27 PE<br>anti-CD38 PE-Vio770<br>anti-IgM APC<br>anti-CD19 APC-Vio770	**CD19 Naïve B cells (NaïveB2):** CD19+CD3-CD27-IgM+<br>**CD19 Memory B cells (MemB2):** CD19+CD27+<br>- **Class Switched Memory B cells (CS MemB):** CD19+CD3-CD27+IgM-<br>- **Non-Class Switched Memory B cells (NoCS MemB):** CD19+CD3-CD27+IgM+<br>**Immature B cells (ImmatB):** CD19+CD3-CD27-IgM-<br>**Transitional B cells (TransitB):** CD19+CD27-CD24^{high}CD38^{high}<br>**Plasmablasts (PB):** CD19+CD3-IgM-CD38^{high}CD24-<br>**Regulatory B cells:**<br>- **RegB1:** CD19+CD3-CD27+CD24high<br>- **RegB2:** CD19+CD27+CD24^{high}CD38^{high}
**5**	anti-CD3 VioGreen<br>anti-CD27 PE<br>anti-CD138 PE-Vio770<br>anti-CD5 APC	**Plasmatic cells (PC):** CD19+CD3-CD27+CD138+<br>**CD5+ B cells:** CD19+CD3-CD5+

	anti-CD19 APC-Vio770	
**6**	Surface staining:<br>anti-CD3 VioGreen<br>anti-CD8 PerCP<br>Intracellular staining:<br>anti-IFNγ FITC<br>anti-IL-17A PE<br>anti-IL-2 APC-Vio770	**IL-2+:** CD3+IL-2+<br>- **IL-2+CD4:** CD3+CD8-IL-2+<br>- **IL-2+CD8:** CD3+CD8+IL-2+<br>**IL-17+:** CD3+IL-17A+<br>- **IL-17+CD4:** CD3+CD8-IL-17A+<br>- **IL-17+CD8:** CD3+CD8+IL-17A+<br>**IFNγ+:** CD3+IFNγ+<br>- **IFNγ+CD4:** CD3+CD8-IFNγ+<br>- **IFNγ+CD8:** CD3+CD8+IFNγ+<br>**IL-17+IFNγ+:** CD3+IL-17A+IFNγ+<br>- **IL-17+IFNγ+CD4:** CD3+CD8-IL-17A+IFNγ+<br>- **IL-17+IFNγ+CD8:** CD3+CD8+IL-17A+IFNγ+
**7**	Surface staining:<br>anti-CD3 VioGreen<br>anti-CD8 PerCP<br>Intracellular staining:<br>anti-IL-4 PE	**IL-4+**: CD3+IL-4+<br>- **IL-4+CD4:** CD3+CD8-IL-4+<br>- **IL-4+CD8:** CD3+CD8+IL-4+
**8**	Surface staining:<br>anti-CD3 VioGreen<br>anti-CD8 PerCP<br>Intracellular staining:<br>anti-IL-22 Vio515	**IL-22+:** CD3+IL-22+<br>- **IL-22+CD4:** CD3+CD8-IL-22+<br>- **IL-22+CD8:** CD3+CD8+IL-22+

Eight panels of fluorochrome-conjugated antibodies were designed to characterize 54 monocyte and lymphocyte subpopulations according to surface and intracellular markers.
