# Supplementary material for: Dimethyl fumarate-related immune and transcriptional signature is associated with clinical response in multiple sclerosis-treated patients
Source: Front Immunol. 2023 Jul 7;14:1209923. doi: 10.3389/fimmu.2023.1209923 (PMC10360655; doi:10.3389/fimmu.2023.1209923)
Supplement: Supplementary file 3 [file DataSheet_3.pdf]

Supplementary Table 2. Differences in monocyte and lymphocyte subpopulations between healthy donors and multiple sclerosis patients

	Percentages†		p-values‡
	HD (n=10)	MS (n=22)	HD vs MS
**Monocytes**	78,62 ± 6,76	83,53 ± 6,08	**0,0433**
**Classical**	63,33 ± 9,73	67,57 ± 7,26	0,4164
**Intermediate**	8,84 ± 3,97	9,39 ± 3,15	0,7872
**Non-classical**	2,45 ± 1,59	2,21 ± 1,65	0,5958
**T lymphocytes**	67,76 ± 8,87	66,71 ± 14,20	0,7643
**Helper T cells**	49,80 ± 7,97	48,76 ± 10,38	0,8888
**Cytotoxic T cells**	14,97 ± 4,40	16,22 ± 6,18	0,5616
**B lymphocytes**	11,99 ± 6,32	15,57 ± 8,99	0,4110
**NKT**	2,53 ± 2,14	3,59 ± 3,37	0,6455
**NK**	12,96 ± 4,97	8,99 ± 5,37	**0,0202**
**Nkbright (% of NK)**	6,35 ± 3,58	14,57 ± 10,84	**0,0069**
**Nkdim (% of NK)**	93,65 ± 3,58	85,43 ± 10,84	**0,0069**
**CD4 TEM**	9,04 ± 3,28	5,98 ± 2,90	**0,0222**
**CD4 TEMRA**	1,29 ± 1,39	1,81 ± 1,46	0,2819
**CD4 TCM**	23,37 ± 4,73	16,75 ± 6,59	**0,0071**
**CD4 Tnaïve**	22,65 ± 5,91	27,54 ± 8,82	0,1512
**CD8 TEM**	4,14 ± 1,54	3,08 ± 1,82	0,0699
**CD8 TEMRA**	7,53 ± 2,25	7,16 ± 5,24	0,4344
**CD8 TCM**	2,45 ± 1,42	2,03 ± 1,82	0,2224
**CD8 Tnaïve**	5,54 ± 2,70	8,63 ± 5,10	0,1168
**RegT**	1,50 ± 0,69	0,76 ± 0,40	**0,0002**
**NaïveB1 (% of CD20+)**	60,84 ± 17,70	76,00 ± 16,26	**0,0311**
**MemB1 (% of CD20+)**	35,82 ± 18,00	20,47 ± 14,85	**0,0175**
**B1 (% of CD20+)**	1,37 ± 0,70	1,62 ± 1,42	0,7264
**B1 CD11b+ (% of CD20+)**	1,31 ± 0,70	0,96 ± 0,96	0,0531
**ImmatB (% of CD19+)**	54,99 ± 15,07	59,68 ± 13,87	0,6455
**NaïveB2 (% of CD19+)**	8,57 ± 3,88	17,96 ± 7,46	**0,0002**
**CS MemB (% of CD19+)**	23,13 ± 7,81	13,00 ± 8,06	**0,0034**
**NoCS MemB (% of CD19+)**	13,31 ± 9,18	9,36 ± 8,28	0,1762
**MemB2 (% of CD19+)**	36,45 ± 14,20	22,36 ± 14,50	**0,0474**
**TransitB (% of CD19+)**	2,30 ± 0,85	1,39 ± 0,83	**0,0137**
**PB (% of CD19+)**	7,65 ± 5,38	4,78 ± 4,68	0,1092
**RegB (% of CD19+)**	26,39 ± 11,38	16,68 ± 12,58	**0,0465**
**RegB2 (% of CD19+)**	2,07 ± 0,91	5,05 ± 7,92	**0,0474**
**PC (% of CD19+)**	1,14 ± 0,67	1,26 ± 1,26	0,5030
**CD5+ B cells (% of CD19+)**	10,08 ± 3,30	9,55 ± 4,71	0,3884
**IL-17+**	0,43 ± 0,22	0,40 ± 0,41	0,3097
**IL-17+CD4**	0,34 ± 0,18	0,35 ± 0,41	0,3399
**IL-17+CD8**	0,04 ± 0,02	0,04 ± 0,03	0,5358
**IFNγ+**	22,03 ± 7,83	14,64 ± 6,18	**0,0071**
**IFNγ+CD4**	11,62 ± 4,17	8,19 ± 3,29	**0,0222**
**IFNγ+CD8**	9,44 ± 4,71	5,45 ± 3,74	**0,0193**
**IL-2+**	11,60 ± 3,49	12,62 ± 5,63	0,5087
**IL-2+CD4**	10,92 ± 3,08	11,52 ± 5,37	0,7271
**IL-2+CD8**	0,96 ± 0,72	1,22 ± 1,05	0,5022
**IL-17+IFNγ+**	0,12 ± 0,08	0,13 ± 0,10	0,7714
**IL-17+IFNγ+CD4**	0,08 ± 0,05	0,08 ± 0,08	0,2618
**IL-17+IFNγ+CD8**	0,04 ± 0,02	0,03 ± 0,02	0,5697
**IL-4+**	0,77 ± 0,38	0,36 ± 0,24	**0,0030**
**IL-4+CD4**	0,59 ± 0,28	0,30 ± 0,20	**0,0059**
**IL-4+CD8**	0,19 ± 0,15	0,08 ± 0,04	**0,0409**
**IL-22+**	0,09 ± 0,05	0,55 ± 0,40	**0,0004**
**IL-22+CD4**	0,07 ± 0,05	0,44 ± 0,36	**0,0007**
**IL-22+CD8**	0,03 ± 0,02	0,07 ± 0,05	**0,0031**

Flow cytometry data from the 54 monocyte and lymphocyte subpopulations analysed in healthy donors (HD) and multiple sclerosis patients (MS) before starting dimethyl fumarate treatment. The percentages of each subpopulation were obtained with respect to live cells or to another subpopulation if specified in parentheses.

†Percentage values are the mean ± standard deviation.

‡p-values were calculated using the Mann-Whitney test to compare differences between HD and MS. $p<0,05$ was considered statistically significant.
