# Supplementary material for: Dimethyl fumarate-related immune and transcriptional signature is associated with clinical response in multiple sclerosis-treated patients
Source: Front Immunol. 2023 Jul 7;14:1209923. doi: 10.3389/fimmu.2023.1209923 (PMC10360655; doi:10.3389/fimmu.2023.1209923)
Supplement: Supplementary file 4 [file DataSheet_4.pdf]

Supplementary Table 3. Comparison of monocyte and lymphocyte subpopulations between naïve and previously treated patients

	Baseline		1 year		Fold change				
	Percentages†		Percentages†		(1 year/Baseline)		p-values‡		
	Previously Treated (n=14)	Naïve (n=8)	Previously Treated (n=14)	Naïve (n=8)	Previously Treated	Naïve	Baseline	1 year	Change¶
**Monocytes**	84,83 ± 5,26	80,95 ± 7,18	83,09 ± 10,49	84,61 ± 6,31	0,98	1,05	0,1718	0,6642	0,4003
**Classical**	68,79 ± 6,31	65,13 ± 8,88	70,31 ± 10,49	70,17 ± 7,86	1,02	1,08	0,2545	0,8154	0,4880
**Intermediate**	10,09 ± 2,26	7,99 ± 4,31	7,18 ± 3,64	8,36 ± 3,72	0,71	1,05	**0,0461**	0,4020	0,0938
**Non-classical**	1,76 ± 1,24	3,11 ± 2,08	1,53 ± 0,90	1,61 ± 0,72	0,87	0,52	0,1056	0,8539	0,1718
**T lymphocytes**	71,15 ± 7,66	58,93 ± 19,68	71,13 ± 11,45	71,02 ± 5,78	1,00	1,21	0,1876	0,4822	0,1266
**Helper T cells**	52,23 ± 4,86	42,69 ± 14,59	56,63 ± 10,85	55,21 ± 9,18	1,08	1,29	0,0950	0,8154	0,1876
**Cytotoxic T cells**	16,99 ± 5,66	14,88 ± 7,20	13,29 ± 5,41	14,47 ± 5,75	0,78	0,97	0,5699	0,3650	0,1932
**B lymphocytes**	14,97 ± 5,97	16,61 ± 13,19	11,70 ± 5,05	10,84 ± 2,55	0,78	0,65	0,4020	>0,9999	0,3650
**NKT**	2,48 ± 1,99	5,55 ± 4,44	1,93 ± 1,89	2,57 ± 1,82	0,78	0,46	0,0817	0,2973	0,0817
**NK**	7,50 ± 3,59	11,59 ± 7,10	10,96 ± 7,81	11,53 ± 3,52	1,46	1,00	0,1450	0,4411	0,2973
**Nkbright (% of NK)**	17,76 ± 12,05	9,01 ± 5,19	18,16 ± 10,38	12,12 ± 8,27	1,02	1,35	**0,0482**	0,1403	0,8676
**Nkdim (% of NK)**	82,24 ± 12,05	90,99 ± 5,19	81,84 ± 10,38	87,88 ± 8,27	1,00	0,97	**0,0482**	0,1403	0,8676
**CD4 TEM**	6,09 ± 3,00	5,78 ± 2,91	3,52 ± 1,47	3,73 ± 3,53	0,58	0,65	0,7135	0,2119	0,7135
**CD4 TEMRA**	1,43 ± 1,34	2,48 ± 1,50	2,03 ± 3,29	1,55 ± 0,97	1,42	0,63	**0,0336**	0,5364	0,3737
**CD4 TCM**	18,40 ± 5,31	13,86 ± 7,94	10,70 ± 5,78	11,15 ± 6,59	0,58	0,80	0,1876	0,7639	0,1653
**CD4 Tnaïve**	29,53 ± 6,76	24,05 ± 11,26	38,81 ± 14,99	41,66 ± 13,29	1,31	1,73	0,1266	0,6163	0,2119
**CD8 TEM**	3,33 ± 2,04	2,65 ± 1,36	1,41 ± 0,99	1,55 ± 1,61	0,42	0,59	0,7135	0,7515	0,8025
**CD8 TEMRA**	6,07 ± 4,93	9,08 ± 5,52	5,93 ± 3,94	5,65 ± 1,81	0,98	0,62	0,1100	0,8154	0,2973
**CD8 TCM**	2,25 ± 2,10	1,64 ± 1,24	0,77 ± 0,73	0,75 ± 0,61	0,34	0,46	0,8154	0,8542	0,4411
**CD8 Tnaïve**	8,92 ± 4,72	8,13 ± 6,00	9,23 ± 5,25	11,47 ± 5,78	1,03	1,41	0,6284	0,3301	0,1228
**RegT**	0,86 ± 0,43	0,57 ± 0,26	0,52 ± 0,27	0,55 ± 0,29	0,61	0,96	0,0616	0,8023	0,1602
**NaïveB1 (% of CD20+)**	81,11 ± 11,83	67,07 ± 19,73	85,45 ± 8,35	77,35 ± 10,52	1,05	1,15	0,1100	0,0502	0,4020
**MemB1 (% of CD20+)**	16,10 ± 11,30	28,12 ± 17,88	9,65 ± 6,86	18,37 ± 9,49	0,60	0,65	0,1100	**0,0103**	0,4411
**B1 (% of CD20+)**	1,24 ± 1,20	2,29 ± 1,62	1,13 ± 0,71	1,42 ± 0,90	0,92	0,62	0,1602	0,5252	0,3650
**B1 CD11b+ (% of CD20+)**	1,06 ± 1,13	0,78 ± 0,58	0,55 ± 0,39	0,54 ± 0,22	0,52	0,69	0,5748	0,5140	0,6988
**ImmatB (% of CD19+)**	63,61 ± 10,19	52,32 ± 14,06	70,42 ± 9,93	57,90 ± 11,37	1,11	1,11	0,0817	**0,0369**	0,6992
**NaïveB2 (% of CD19+)**	16,33 ± 6,48	15,12 ± 5,58	17,90 ± 6,87	21,73 ± 8,65	1,10	1,44	0,6163	0,4854	0,0557
**CS MemB (% of CD19+)**	12,43 ± 7,77	18,99 ± 7,33	7,47 ± 3,63	11,00 ± 3,85	0,60	0,58	0,0502	0,0531	0,1827
**NoCS MemB (% of CD19+)**	7,59 ± 6,27	13,57 ± 9,78	4,21 ± 4,55	9,37 ± 7,25	0,55	0,69	0,1063	0,0557	0,5356
**MemB2 (% of CD19+)**	23,26 ± 12,93	32,56 ± 14,76	11,68 ± 7,26	20,37 ± 9,12	0,50	0,63	0,1266	**0,0186**	0,7573
**TransitB (% of CD19+)**	71,06 ± 17,49	63,02 ± 13,42	83,46 ± 12,65	75,17 ± 6,60	1,17	1,19	0,1266	0,0968	0,8168
**PB (% of CD19+)**	3,51 ± 3,96	2,27 ± 1,50	2,25 ± 2,06	3,43 ± 2,84	0,64	1,52	0,8803	0,2749	0,3824
**RegB (% of CD19+)**	13,42 ± 9,05	23,19 ± 13,07	7,27 ± 6,17	14,97 ± 7,40	0,54	0,65	0,0698	**0,0369**	0,3507
**RegB2 (% of CD19+)**	3,04 ± 1,65	3,28 ± 2,10	2,28 ± 1,32	2,37 ± 0,70	0,75	0,72	0,9734	0,5356	>0,9999
**PC (% of CD19+)**	0,99 ± 1,04	1,72 ± 1,53	1,19 ± 1,19	1,91 ± 1,78	1,20	1,11	0,4113	**0,0366**	0,9734
**CD5+ B cells (% of CD19+)**	8,62 ± 3,49	11,16 ± 6,26	14,25 ± 5,38	12,84 ± 6,02	1,65	1,15	0,4822	0,3301	0,1100
**IL-17+**	0,45 ± 0,49	0,31 ± 0,21	0,23 ± 0,08	0,26 ± 0,09	0,52	0,82	0,1596	0,3917	0,1598
**IL-17+CD4**	0,40 ± 0,50	0,26 ± 0,13	0,18 ± 0,06	0,24 ± 0,10	0,44	0,91	0,5358	0,2885	0,3215
**IL-17+CD8**	0,04 ± 0,02	0,05 ± 0,03	0,04 ± 0,02	0,04 ± 0,02	0,97	0,73	0,4278	0,9038	0,4682
**IFNγ+**	14,37 ± 5,87	15,11 ± 7,08	4,56 ± 2,83	5,90 ± 2,77	0,32	0,39	0,8676	0,0673	0,8154
**IFNγ+CD4**	7,86 ± 2,96	8,75 ± 3,96	2,64 ± 1,49	3,83 ± 2,23	0,34	0,44	0,9734	0,1450	0,6163
**IFNγ+CD8**	5,48 ± 3,87	5,40 ± 3,77	1,79 ± 1,54	1,80 ± 0,78	0,33	0,33	0,8806	0,3219	0,7639
**IL-2+**	13,95 ± 4,57	10,28 ± 6,82	8,65 ± 4,78	6,19 ± 3,67	0,62	0,60	0,1653	0,4411	0,6163
**IL-2+CD4**	12,87 ± 4,51	9,16 ± 6,23	7,73 ± 4,21	5,57 ± 3,93	0,60	0,61	0,1931	0,3650	0,7639
**IL-2+CD8**	1,47 ± 1,16	0,79 ± 0,69	0,57 ± 0,36	0,37 ± 0,24	0,39	0,47	0,0985	0,1703	0,1137
**IL-17+IFNγ+**	0,12 ± 0,12	0,15 ± 0,08	0,09 ± 0,03	0,14 ± 0,09	0,77	0,90	0,1372	0,2728	0,7503
**IL-17+IFNγ+CD4**	0,07 ± 0,10	0,08 ± 0,04	0,08 ± 0,06	0,05 ± 0,04	1,12	0,62	0,0645	0,2546	0,0604
**IL-17+IFNγ+CD8**	0,03 ± 0,02	0,04 ± 0,03	0,04 ± 0,02	0,03 ± 0,02	1,40	0,91	0,5140	0,4912	0,3047
**IL-4+**	0,45 ± 0,23	0,21 ± 0,17	0,25 ± 0,15	0,23 ± 0,16	0,55	1,12	**0,0052**	0,9336	**0,0402**
**IL-4+CD4**	0,35 ± 0,20	0,21 ± 0,19	0,19 ± 0,14	0,17 ± 0,14	0,56	0,80	0,1056	0,4709	0,2891
**IL-4+CD8**	0,10 ± 0,04	0,05 ± 0,03	0,05 ± 0,04	0,04 ± 0,02	0,54	0,70	**0,0144**	0,4915	0,1207
**IL-22+**	0,50 ± 0,44	0,62 ± 0,33	0,51 ± 0,33	0,54 ± 0,34	1,02	0,88	0,3545	0,8550	0,5814
**IL-22+CD4**	0,41 ± 0,37	0,50 ± 0,36	0,42 ± 0,28	0,45 ± 0,31	1,02	0,91	0,6761	0,9071	0,6045
**IL-22+CD8**	0,07 ± 0,05	0,09 ± 0,05	0,09 ± 0,07	0,06 ± 0,05	1,37	0,71	0,2551	0,3199	0,1119

Flow cytometry data from the 54 monocyte and lymphocyte subpopulations analysed in naïve and previously treated multiple sclerosis patients at baseline and after 1 year of dimethyl fumarate treatment. The percentages of each subpopulation were obtained with respect to live cells or to another subpopulation if specified in parentheses.

†Percentage values are the mean ± standard deviation.

‡p-values were calculated using the Mann-Whitney test to compare differences between naïve and previously treated patients at baseline, at 1 year and for the change between both timepoints. $p<0,05$ was considered statistically significant.

¶The differences in the percentages at baseline minus the percentages at 1 year were calculated for naïve and previously treated patients.
