# Supplementary material for: Dimethyl fumarate-related immune and transcriptional signature is associated with clinical response in multiple sclerosis-treated patients
Source: Front Immunol. 2023 Jul 7;14:1209923. doi: 10.3389/fimmu.2023.1209923 (PMC10360655; doi:10.3389/fimmu.2023.1209923)
Supplement: Supplementary file 5 [file DataSheet_5.pdf]

Supplementary Table 4. Dimethyl fumarate effects on monocyte and lymphocyte subpopulations after 1 year of treatment

	Percentages†		Fold change	p-values‡
	Baseline (n=22)	1 year (n=22)	1 year/Baseline	Baseline vs 1 year
**Monocytes**	83,53 ± 6,08	83,64 ± 9,06	1,00	0,7335
**Classical**	67,57 ± 7,26	70,26 ± 9,42	1,04	0,3162
**Intermediate**	9,39 ± 1,65	7,61 ± 0,82	0,71	0,0822
**Non-classical**	2,21 ± 3,15	1,56 ± 3,63	0,81	0,1937
**T lymphocytes**	66,71 ± 14,20	71,09 ± 9,61	1,07	0,2650
**Helper T cells**	48,76 ± 10,38	56,12 ± 10,07	1,15	**0,0066**
**Cytotoxic T cells**	16,22 ± 6,18	13,72 ± 5,43	0,85	**0,0008**
**B lymphocytes**	15,57 ± 8,99	11,39 ± 4,26	0,73	**0,0229**
**NKT**	3,59 ± 3,37	2,17 ± 1,85	0,60	**0,0005**
**NK**	8,99 ± 5,37	11,17 ± 6,47	1,24	**0,0462**
**Nkbright (% of NK)**	14,57 ± 10,84	15,96 ± 9,92	1,10	0,1207
**Nkdim (% of NK)**	85,43 ± 10,84	84,04 ± 9,92	0,98	0,1207
**CD4 TEM**	5,98 ± 2,90	3,60 ± 2,34	0,60	**0,0002**
**CD4 TEMRA**	1,81 ± 1,46	1,85 ± 2,66	1,02	0,4106
**CD4 TCM**	16,75 ± 6,59	10,86 ± 5,93	0,65	**0,0017**
**CD4 Tnaïve**	27,54 ± 8,82	39,85 ± 14,14	1,45	**0,0002**
**CD8 TEM**	3,08 ± 1,82	1,46 ± 1,21	0,47	**<0,0001**
**CD8 TEMRA**	7,16 ± 5,24	5,83 ± 3,27	0,81	0,2348
**CD8 TCM**	2,03 ± 1,82	0,76 ± 0,67	0,38	**0,0001**
**CD8 Tnaïve**	8,63 ± 5,10	10,04 ± 5,42	1,16	0,0535
**RegT**	0,76 ± 0,40	0,53 ± 0,27	0,70	**0,0351**
**NaïveB1 (% of CD20+)**	76,00 ± 16,26	82,50 ± 9,79	1,09	0,0587
**MemB1 (% of CD20+)**	20,47 ± 14,85	12,82 ± 8,81	0,63	**0,0275**
**B1 (% of CD20+)**	1,62 ± 1,42	1,24 ± 0,78	0,76	0,5080
**B1 CD11b+ (% of CD20+)**	0,96 ± 0,96	0,54 ± 0,33	0,57	**0,0266**
**ImmatB (% of CD19+)**	59,68 ± 13,87	65,33 ± 11,32	1,09	0,0696
**NaïveB2 (% of CD19+)**	17,96 ± 7,46	20,14 ± 7,30	1,12	0,1621
**CS MemB (% of CD19+)**	13,00 ± 8,06	8,52 ± 3,98	0,66	**0,0136**
**NoCS MemB (% of CD19+)**	9,36 ± 8,28	5,95 ± 6,06	0,64	**0,0484**
**MemB2 (% of CD19+)**	22,36 ± 14,50	14,47 ± 8,85	0,65	**0,0215**
**TransitB (% of CD19+)**	1,39 ± 0,83	1,37 ± 0,94	0,98	0,8408
**PB (% of CD19+)**	4,78 ± 4,68	6,12 ± 7,43	1,28	0,7771
**RegB (% of CD19+)**	16,68 ± 12,58	10,46 ± 7,74	0,63	**0,0362**
**RegB2 (% of CD19+)**	5,05 ± 7,92	1,79 ± 1,04	0,35	**0,0083**
**PC (% of CD19+)**	1,26 ± 1,26	1,45 ± 1,43	1,15	0,6556
**CD5+ B cells (% of CD19+)**	9,55 ± 4,71	13,73 ± 5,52	1,44	**0,0019**
**IL-17+**	0,40 ± 0,41	0,24 ± 0,08	0,60	**0,0051**
**IL-17+CD4**	0,35 ± 0,41	0,20 ± 0,08	0,57	**0,0044**
**IL-17+CD8**	0,04 ± 0,03	0,04 ± 0,02	0,88	0,5630
**IFNγ+**	14,64 ± 6,18	5,04 ± 2,82	0,34	**<0,0001**
**IFNγ+CD4**	8,19 ± 3,29	3,07 ± 1,84	0,38	**<0,0001**
**IFNγ+CD8**	5,45 ± 3,74	1,80 ± 1,29	0,33	**<0,0001**
**IL-2+**	12,62 ± 5,63	7,75 ± 4,48	0,61	**0,0037**
**IL-2+CD4**	11,52 ± 5,37	6,94 ± 4,15	0,60	**0,0059**
**IL-2+CD8**	1,22 ± 1,05	0,50 ± 0,33	0,41	**<0,0001**
**IL-17+IFNγ+**	0,13 ± 0,10	0,11 ± 0,06	0,83	0,4157
**IL-17+IFNγ+CD4**	0,08 ± 0,08	0,07 ± 0,06	0,92	0,5381
**IL-17+IFNγ+CD8**	0,03 ± 0,02	0,04 ± 0,02	1,19	0,3963
**IL-4+**	0,36 ± 0,24	0,24 ± 0,15	0,67	**0,0470**
**IL-4+CD4**	0,30 ± 0,20	0,19 ± 0,14	0,62	**0,0268**
**IL-4+CD8**	0,08 ± 0,04	0,05 ± 0,03	0,58	**0,0072**
**IL-22+**	0,55 ± 0,40	0,52 ± 0,32	0,96	0,7563
**IL-22+CD4**	0,44 ± 0,36	0,43 ± 0,29	0,97	0,8175
**IL-22+CD8**	0,07 ± 0,05	0,08 ± 0,06	1,09	0,8191

Flow cytometry data from the 54 monocyte and lymphocyte subpopulations analysed in multiple sclerosis patients at baseline and after 1 year of dimethyl fumarate treatment. The percentages of each subpopulation were obtained with respect to live cells or to another subpopulation if specified in parentheses.

†Percentage values are the mean ± standard deviation.

‡p-values were calculated using the Wilcoxon signed-rank test to compare differences between the percentages at baseline and at 1 year. $p<0,05$ was considered statistically significant.
