# Supplementary material for: Dimethyl fumarate-related immune and transcriptional signature is associated with clinical response in multiple sclerosis-treated patients
Source: Front Immunol. 2023 Jul 7;14:1209923. doi: 10.3389/fimmu.2023.1209923 (PMC10360655; doi:10.3389/fimmu.2023.1209923)
Supplement: Supplementary file 6 [file DataSheet_6.pdf]

Supplementary Table 5. Comparison of monocyte and lymphocyte subpopulations between NEDA-3 and EDA-3 patients

	Baseline Percentages†		1 year Percentages		Fold change (1 year/Baseline)		p-values‡		
	**NEDA-3 (n=15)**	**EDA-3 (n=7)**	**NEDA-3 (n=15)**	**EDA-3 (n=7)**	**NEDA-3 (n=15)**	**EDA-3 (n=7)**	**Baseline**	**1 year**	**Change¶**
**Monocytes**	83,61 ± 6,01	83,38 ± 6,69	84,79 ± 6,01	81,18 ± 13,87	1,01	0,97	0,9710	0,8907	0,8557
**Classical**	68,01 ± 6,76	66,68 ± 8,68	70,12 ± 7,14	70,54 ± 13,82	1,03	1,06	0,8557	0,5349	0,7990
**Intermediate**	9,07 ± 2,60	10,01 ± 4,21	8,86 ± 3,74	4,93 ± 1,03	0,74	0,64	0,8557	**0,0021**	**0,0461**
**Non-classical**	2,21 ± 1,70	2,21 ± 1,67	1,63 ± 0,86	1,41 ± 0,78	0,98	0,49	>0,9999	0,6674	0,6359
**T lymphocytes**	70,20 ± 7,88	59,22 ± 21,56	70,13 ± 10,61	73,16 ± 7,28	1,00	1,24	0,4902	0,7833	0,1229
**Helper T cells**	50,48 ± 5,71	45,08 ± 16,66	54,33 ± 9,21	59,94 ± 11,50	1,08	1,33	0,8907	0,2982	0,1624
**Cytotoxic T cells**	17,74 ± 5,66	12,95 ± 6,39	14,40 ± 5,52	12,27 ± 5,33	0,81	0,95	0,1229	0,4069	0,8242
**B lymphocytes**	13,64 ± 6,51	19,69 ± 12,44	11,74 ± 4,82	10,63 ± 2,88	0,86	0,54	0,3684	0,9452	0,2666
**NKT**	3,58 ± 3,35	3,62 ± 3,67	2,14 ± 1,89	2,22 ± 1,90	0,60	0,61	0,8907	0,8907	>0,9999
**NK**	7,32 ± 3,37	12,55 ± 7,27	11,80 ± 7,05	9,80 ± 5,24	1,61	0,78	0,0659	0,7309	**0,0387**
**Nkbright (% of NK)**	17,10 ± 10,88	9,16 ± 9,19	17,03 ± 9,55	13,65 ± 11,06	1,00	1,49	**0,0164**	0,2596	0,6796
**Nkdim (% of NK)**	82,90 ± 10,88	90,84 ± 9,19	82,97 ± 9,55	86,35 ± 11,06	1,00	0,95	**0,0164**	0,2596	0,6796
**CD4 TEM**	6,73 ± 3,01	4,35 ± 1,97	3,24 ± 1,48	4,37 ± 3,62	0,48	1,00	0,0777	0,6796	**0,0029**
**CD4 TEMRA**	1,74 ± 1,36	1,96 ± 1,77	1,05 ± 0,72	3,58 ± 4,29	0,60	1,83	0,9326	**0,0064**	0,2160
**CD4 TCM**	17,93 ± 5,57	14,21 ± 8,29	12,48 ± 6,34	7,39 ± 2,94	0,70	0,52	0,2666	**0,0465**	>0,9999
**CD4 Tnaïve**	27,47 ± 8,07	27,67 ± 10,98	39,26 ± 12,49	41,11 ± 18,23	1,43	1,49	>0,9999	0,5815	0,7833
**CD8 TEM**	3,56 ± 1,83	2,07 ± 1,43	1,46 ± 0,93	1,47 ± 1,77	0,41	0,71	0,0556	0,4159	**0,0162**
**CD8 TEMRA**	6,68 ± 5,16	8,19 ± 5,65	6,40 ± 3,43	4,62 ± 2,72	0,96	0,56	0,5815	0,3684	0,2372
**CD8 TCM**	2,45 ± 2,03	1,12 ± 0,80	0,89 ± 0,75	0,48 ± 0,40	0,36	0,43	**0,0387**	0,1678	0,1624
**CD8 Tnaïve**	9,12 ± 5,03	7,60 ± 5,47	10,43 ± 5,67	9,22 ± 5,17	1,14	1,21	0,4572	0,8366	0,2909
**RegT**	0,77 ± 0,46	0,73 ± 0,25	0,62 ± 0,27	0,36 ± 0,16	0,80	0,49	0,9041	**0,0480**	0,2908
**NaïveB1 (% of CD20+)**	76,25 ± 19,12	75,49 ± 8,44	82,23 ± 11,02	83,08 ± 7,19	1,08	1,10	0,5349	0,6298	>0,9999
**MemB1 (% of CD20+)**	20,49 ± 17,67	20,42 ± 6,61	13,11 ± 9,67	12,20 ± 7,25	0,64	0,60	0,4902	0,8366	0,8907
**B1 (% of CD20+)**	1,57 ± 1,45	1,72 ± 1,47	1,24 ± 0,74	1,22 ± 0,91	0,79	0,71	0,6913	0,6298	0,8366
**B1 CD11b+ (% of CD20+)**	0,82 ± 0,52	1,22 ± 1,51	0,59 ± 0,39	0,45 ± 0,15	0,72	0,37	0,8318	0,5694	0,5350
**ImmatB (% of CD19+)**	59,92 ± 15,10	59,18 ± 11,88	64,68 ± 12,63	66,54 ± 9,17	1,08	1,12	>0,9999	0,9385	0,7573
**NaïveB2 (% of CD19+)**	18,61 ± 7,48	16,57 ± 7,81	19,95 ± 7,50	20,51 ± 7,48	1,07	1,24	0,5815	0,5356	0,7132
**CS MemB (% of CD19+)**	12,22 ± 9,57	14,67 ± 3,00	8,46 ± 3,60	8,65 ± 4,92	0,69	0,59	0,4475	0,8773	>0,9999
**NoCS MemB (% of CD19+)**	9,26 ± 9,53	9,59 ± 5,27	6,84 ± 7,15	4,30 ± 3,01	0,74	0,45	0,5349	0,8018	0,6992
**MemB2 (% of CD19+)**	21,48 ± 17,11	24,25 ± 6,82	15,30 ± 9,70	12,95 ± 7,46	0,71	0,53	0,5815	0,8773	0,7573
**TransitB (% of CD19+)**	1,22 ± 0,69	1,77 ± 1,01	1,25 ± 0,89	1,59 ± 1,08	1,03	0,90	0,2666	0,3507	0,6992
**PB (% of CD19+)**	5,60 ± 5,34	3,03 ± 2,22	5,35 ± 4,16	7,55 ±11,68	0,96	2,49	0,4069	0,5356	0,6853
**RegB (% of CD19+)**	16,13 ± 14,85	17,86 ± 6,08	10,93 ± 8,22	9,56 ± 7,28	0,68	0,54	0,4376	>0,9999	0,7573
**RegB2 (% of CD19+)**	6,38 ± 9,36	2,21 ± 1,00	2,07 ± 1,15	1,29 ± 0,58	0,32	0,58	0,0556	0,2414	0,3114
**PC (% of CD19+)**	1,05 ± 0,99	1,70 ± 1,71	1,39 ± 1,11	1,57 ± 2,07	1,33	0,92	0,4379	0,5705	0,4475
**CD5+ B cells (% of CD19+)**	8,43 ± 3,97	11,93 ± 5,58	12,67 ± 4,70	16,02 ± 6,80	1,50	1,34	0,1624	0,2666	0,7309
**IL-17+**	0,45 ± 0,48	0,30 ± 0,13	0,25 ± 0,08	0,23 ± 0,08	0,55	0,78	0,5692	0,6915	0,5233
**IL-17+CD4**	0,39 ± 0,49	0,26 ± 0,11	0,20 ± 0,09	0,20 ± 0,08	0,51	0,74	0,7178	0,6664	0,7961
**IL-17+CD8**	0,05 ± 0,03	0,04 ± 0,02	0,04 ± 0,02	0,03 ± 0,02	0,87	0,90	0,4668	0,4134	0,8222
**IFNγ+**	15,56 ± 6,73	12,68 ± 4,60	5,04 ± 2,63	5,05 ± 3,40	0,32	0,40	0,5349	0,9034	0,4902
**IFNγ+CD4**	8,74 ± 3,31	6,99 ± 3,15	2,95 ± 1,39	3,34 ± 2,67	0,34	0,48	0,2982	0,8366	0,1417
**IFNγ+CD8**	6,07 ± 4,03	4,12 ± 2,83	1,92 ± 1,45	1,54 ± 0,91	0,32	0,37	0,3978	0,6914	0,2982
**IL-2+**	13,23 ± 5,16	11,31 ± 6,78	8,31 ± 4,61	6,56 ± 4,27	0,63	0,58	0,4475	0,4069	0,8366
**IL-2+CD4**	12,15 ± 4,94	10,16 ± 6,39	7,43 ± 4,27	5,89 ± 3,99	0,61	0,58	0,4571	0,4069	0,7309
**IL-2+CD8**	1,46 ± 1,16	0,71 ± 0,53	0,58 ± 0,37	0,32 ± 0,13	0,40	0,45	0,0874	0,075	0,2587
**IL-17+IFNγ+**	0,13 ± 0,11	0,13 ± 0,09	0,11 ± 0,05	0,11 ± 0,08	0,82	0,84	0,6404	0,4572	0,742
**IL-17+IFNγ+CD4**	0,08 ± 0,09	0,07 ± 0,05	0,06 ± 0,05	0,09 ± 0,07	0,75	1,35	0,688	0,4968	0,3753
**IL-17+IFNγ+CD8**	0,03 ± 0,02	0,03 ± 0,03	0,04 ± 0,02	0,03 ± 0,03	1,27	1,01	0,9499	0,2498	0,9046
**IL-4+**	0,33 ± 0,22	0,42 ± 0,29	0,26 ± 0,14	0,21 ± 0,17	0,77	0,49	0,4362	0,3217	0,1911
**IL-4+CD4**	0,24 ± 0,16	0,41 ± 0,24	0,19 ± 0,12	0,17 ± 0,19	0,79	0,41	0,1266	0,2894	0,0629
**IL-4+CD8**	0,09 ± 0,05	0,07 ± 0,03	0,05 ± 0,03	0,04 ± 0,04	0,59	0,56	0,6921	0,2199	0,9573
**IL-22+**	0,51 ± 0,39	0,63 ± 0,44	0,50 ± 0,37	0,57 ± 0,18	0,98	0,92	0,6162	0,6415	0,9868
**IL-22+CD4**	0,42 ± 0,34	0,50 ± 0,42	0,42 ± 0,33	0,46 ± 0,18	1,00	0,92	0,6169	0,6918	0,9322
**IL-22+CD8**	0,07 ± 0,05	0,08 ± 0,06	0,07 ± 0,06	0,10 ± 0,07	1,05	1,15	0,6157	0,6411	0,7687

Flow cytometry data from the 54 monocyte and lymphocyte subpopulations analysed in NEDA-3 and EDA-3 multiple sclerosis patients at baseline and after 1 year of dimethyl fumarate treatment. The percentages of each subpopulation were obtained with respect to live cells or to another subpopulation if specified in parentheses.

†Percentage values are the mean ± standard deviation.

‡p-values were calculated using the Mann-Whitney test to compare differences between NEDA-3 and EDA-3 patients at baseline, at 1 year and for the change between both timepoints. $p<0,05$ was considered statistically significant.

¶The differences in the percentages at baseline minus the percentages at 1 year were calculated for NEDA-3 and EDA-3 patients.
