# Supplementary material for: Dimethyl fumarate-related immune and transcriptional signature is associated with clinical response in multiple sclerosis-treated patients
Source: Front Immunol. 2023 Jul 7;14:1209923. doi: 10.3389/fimmu.2023.1209923 (PMC10360655; doi:10.3389/fimmu.2023.1209923)
Supplement: Supplementary file 7 [file DataSheet_7.pdf]

Supplementary Table 6. Comparison of monocyte and lymphocyte subpopulations between female and male patients

	Baseline Percentages†		1 year Percentages†		Fold change (1 year/Baseline)		p-values‡		
	**Women (n=17)**	**Men (n=5)**	**Women (n=17)**	**Men (n=5)**	**Women**	**Men**	**Baseline**	**1 year**	**Change¶**
**Monocytes**	82,53 ± 6,23	86,76 ± 4,69	84,68 ± 7,45	80,12 ± 13,72	1,03	0,92	0,1530	0,6486	0,2748
**Classical**	65,93 ± 6,90	72,82 ± 6,32	70,57 ± 8,61	69,18 ± 12,92	1,07	0,95	0,0753	>0,9999	0,2080
**Intermediate**	9,40 ± 3,20	9,33 ± 3,34	8,19 ± 3,86	5,66 ± 1,84	0,87	0,61	0,8417	0,0849	0,3539
**Non-classical**	2,60 ± 1,68	0,95 ± 0,59	1,64 ± 0,84	1,29 ± 0,78	0,63	1,36	**0,0137**	0,4543	0,0500
**T lymphocytes**	69,39 ± 14,06	57,57 ± 11,52	72,52 ± 9,04	66,24 ± 10,93	1,05	1,15	**0,0063**	0,2488	0,5426
**Helper T cells**	49,80 ± 10,22	45,22 ± 11,26	56,30 ± 9,16	55,50 ± 14,02	1,13	1,23	0,5426	0,7043	0,9396
**Cytotoxic T cells**	17,72 ± 6,12	11,12 ± 2,93	14,91 ± 5,12	9,69 ± 4,85	0,84	0,87	**0,0150**	0,0583	0,1846
**B lymphocytes**	12,91 ± 7,25	24,62 ± 9,00	10,65 ± 4,25	13,88 ± 3,58	0,83	0,56	**0,0086**	0,0849	**0,0086**
**NKT**	3,58 ± 3,19	3,64 ± 4,33	2,09 ± 1,65	2,45 ± 2,63	0,58	0,67	0,8795	>0,9999	0,4008
**NK**	8,70 ± 5,84	9,97 ± 3,66	10,98 ± 6,70	11,80 ± 6,31	1,26	1,18	0,1404	0,3587	0,8795
**Nkbright (% of NK)**	15,16 ± 12,10	12,58 ± 4,91	16,49 ± 10,21	14,15 ± 9,70	1,09	1,12	0,7726	0,7726	0,2488
**Nkdim (% of NK)**	84,84 ± 12,10	87,42 ± 4,91	83,51 ± 10,21	85,85 ± 9,70	0,98	0,98	0,8911	0,7726	0,2488
**CD4 TEM**	6,52 ± 3,06	4,13 ± 1,12	3,76 ± 2,57	3,05 ± 1,38	0,58	0,74	0,1012	0,7043	0,2177
**CD4 TEMRA**	1,74 ± 1,36	2,07 ± 1,92	1,38 ± 0,92	3,47 ± 5,42	0,80	1,68	0,9278	0,9514	0,6939
**CD4 TCM**	16,72 ± 5,88	16,82 ± 9,49	11,09 ± 6,35	10,10 ± 4,77	0,66	0,60	0,9396	>0,9999	0,8201
**CD4 Tnaïve**	28,18 ± 9,78	25,33 ± 4,31	42,23 ± 13,29	31,75 ± 15,38	1,50	1,25	0,4929	0,1404	0,1636
**CD8 TEM**	3,15 ± 1,93	2,86 ± 1,56	1,64 ± 1,33	0,85 ± 0,27	0,52	0,30	0,6486	0,3262	0,3894
**CD8 TEMRA**	7,07 ± 4,65	7,47 ± 7,56	5,52 ± 2,65	6,89 ± 5,12	0,78	0,92	0,6486	0,5946	0,4456
**CD8 TCM**	2,25 ± 1,99	1,25 ± 0,79	0,87 ± 0,72	0,38 ± 0,33	0,39	0,31	0,1893	0,1349	0,7043
**CD8 Tnaïve**	9,56 ± 5,10	5,49 ± 4,05	11,05 ± 5,19	6,62 ± 5,25	1,16	1,21	0,1163	0,1012	0,8314
**RegT**	0,70 ± 0,24	0,94 ± 0,73	0,56 ± 0,27	0,44 ± 0,28	0,79	0,47	0,8941	0,4364	0,7726
**NaïveB1 (% of CD20+)**	75,19 ± 17,24	78,77 ± 13,63	80,99 ± 10,26	87,65 ± 6,28	1,08	1,11	0,8795	0,1404	0,8795
**MemB1 (% of CD20+)**	20,84 ± 15,67	19,20 ± 13,13	14,26 ± 9,25	7,93 ± 5,12	0,68	0,41	>0,9999	0,1196	0,7043
**B1 (% of CD20+)**	1,88 ± 1,53	0,74 ± 0,19	1,33 ± 0,83	0,93 ± 0,49	0,71	1,25	0,1942	0,4008	0,5946
**B1 CD11b+ (% of CD20+)**	1,10 ± 1,07	0,55 ± 0,36	0,58 ± 0,35	0,41 ± 0,24	0,53	0,74	0,2735	0,2739	0,3050
**ImmatB (% of CD19+)**	59,01 ± 13,57	61,96 ± 16,30	63,55 ± 12,10	70,67 ± 6,95	1,08	1,14	0,7616	0,1974	0,7354
**NaïveB2 (% of CD19+)**	17,54 ± 7,13	19,38 ± 9,26	20,33 ± 7,37	19,57 ± 7,92	1,16	1,01	0,8201	>0,9999	0,3135
**CS MemB (% of CD19+)**	14,22 ± 8,25	8,84 ± 6,39	9,48 ± 3,98	5,67 ± 2,48	0,67	0,64	0,2177	**0,0418**	0,5531
**NoCS MemB (% of CD19+)**	9,23 ± 8,33	9,81 ± 9,07	6,57 ± 6,35	4,09 ± 5,24	0,71	0,42	>0,9999	0,2034	0,5531
**MemB2 (% of CD19+)**	23,45 ± 14,72	18,65 ± 14,65	16,05 ± 8,89	9,76 ± 7,65	0,68	0,52	0,6486	0,0806	0,8660
**TransitB (% of CD19+)**	1,40 ± 0,80	1,34 ± 1,01	1,32 ± 0,83	1,50 ± 1,33	0,94	1,12	0,6486	>0,9999	0,8001
**PB (% of CD19+)**	4,42 ± 4,21	6,01 ± 6,46	5,44 ± 4,11	8,19 ± 13,99	1,23	1,36	0,7043	0,4973	0,5085
**RegB (% of CD19+)**	17,37 ± 13,12	14,36 ± 11,54	12,04 ± 7,84	5,70 ± 5,59	0,69	0,40	0,6591	**0,0418**	0,7354
**RegB2 (% of CD19+)**	5,76 ± 8,89	2,66 ± 2,04	1,85 ± 1,00	1,62 ± 1,28	0,32	0,61	0,3193	0,3949	0,4973
**PC (% of CD19+)**	1,48 ± 1,35	0,49 ± 0,30	1,44 ± 1,48	1,48 ± 1,41	0,97	3,01	**0,0493**	>0,9999	0,1636
**CD5+ B cells (% of CD19+)**	9,43 ± 5,24	9,94 ± 2,51	12,77 ± 4,89	17,01 ± 6,86	1,35	1,71	0,5426	0,1404	0,4008
**IL-17+**	0,43 ± 0,46	0,31 ± 0,08	0,24 ± 0,09	0,26 ± 0,04	0,55	0,85	0,9254	0,4329	0,5553
**IL-17+CD4**	0,38 ± 0,46	0,25 ± 0,08	0,20 ± 0,09	0,21 ± 0,07	0,52	0,85	0,6621	0,6638	0,1955
**IL-17+CD8**	0,05 ± 0,03	0,04 ± 0,01	0,04 ± 0,02	0,05 ± 0,02	0,79	1,24	0,9798	0,2507	0,2068
**IFNγ+**	15,09 ± 6,59	13,12 ± 4,79	5,11 ± 3,13	4,83 ± 1,58	0,34	0,37	0,7616	0,8314	0,7616
**IFNγ+CD4**	8,48 ± 3,40	7,18 ± 3,02	3,21 ± 2,06	2,61 ± 0,62	0,38	0,36	0,3193	0,7043	0,7616
**IFNγ+CD8**	5,48 ± 3,90	5,33 ± 3,54	1,72 ± 1,11	2,05 ± 1,93	0,31	0,39	0,868	0,8680	>0,9999
**IL-2+**	12,24 ± 4,70	13,89 ± 8,68	6,53 ± 3,77	11,90 ± 4,58	0,53	0,86	0,5426	**0,0387**	0,2488
**IL-2+CD4**	11,10 ± 4,24	12,95 ± 8,73	5,97 ± 3,79	10,23 ± 3,95	0,54	0,79	0,5331	0,1012	0,3587
**IL-2+CD8**	1,38 ± 1,14	0,69 ± 0,47	0,49 ± 0,35	0,52 ± 0,28	0,35	0,75	0,1455	0,6934	**0,0453**
**IL-17+IFNγ+**	0,14 ± 0,12	0,10 ± 0,02	0,12 ± 0,07	0,08 ± 0,04	0,83	0,81	0,839	0,2229	0,6029
**IL-17+IFNγ+CD4**	0,08 ± 0,09	0,05 ± 0,01	0,07 ± 0,06	0,05 ± 0,02	0,92	0,92	0,7453	0,8546	0,8628
**IL-17+IFNγ+CD8**	0,03 ± 0,02	0,04 ± 0,03	0,04 ± 0,02	0,03 ± 0,03	1,29	0,89	0,6995	0,3265	0,4833
**IL-4+**	0,30 ± 0,21	0,57 ± 0,22	0,20 ± 0,12	0,38 ± 0,16	0,68	0,65	**0,0294**	**0,0122**	0,4115
**IL-4+CD4**	0,25 ± 0,18	0,47 ± 0,21	0,16 ± 0,11	0,29 ± 0,19	0,63	0,61	**0,0183**	0,1053	0,5040
**IL-4+CD8**	0,08 ± 0,05	0,08 ± 0,03	0,04 ± 0,02	0,07 ± 0,06	0,49	0,87	0,6902	0,6038	0,5817
**IL-22+**	0,45 ± 0,33	0,86 ± 0,49	0,52 ± 0,35	0,54 ± 0,24	1,14	0,62	0,0965	0,9269	0,0824
**IL-22+CD4**	0,36 ± 0,30	0,73 ± 0,44	0,43 ± 0,31	0,45 ± 0,23	1,19	0,61	0,0739	0,9244	0,2231
**IL-22+CD8**	0,07 ± 0,05	0,08 ± 0,06	0,08 ± 0,06	0,09 ± 0,06	1,07	1,14	0,7694	0,5812	0,7745

Flow cytometry data from the 54 monocyte and lymphocyte subpopulations analysed in female and male multiple sclerosis patients at baseline and after 1 year of dimethyl fumarate treatment. The percentages of each subpopulation were obtained with respect to live cells or to another subpopulation if specified in parentheses.

†Percentage values are the mean ± standard deviation.

‡p-values were calculated using the Mann-Whitney test to compare differences between women and men at baseline, at 1 year and for the change between both timepoints. p<0,05 was considered statistically significant.

¶The differences in the percentages at baseline minus the percentages at 1 year were calculated for women and men.
